# *WWOX*-rs13338697 genotype predicts therapeutic efficacy of ADI-PEG 20 for patients with advanced hepatocellular carcinoma

**DOI:** 10.3389/fonc.2022.996820

**Published:** 2022-12-02

**Authors:** Yu-De Chu, Hui-Fen Liu, Yi-Chen Chen, Chun-Hung Chou, Chau-Ting Yeh

**Affiliations:** ^1^ Liver Research Center, Chang Gung Memorial Hospital, Taoyuan, Taiwan; ^2^ Polaris Pharmaceuticals, Inc., Polaris Group, Taipei, Taiwan; ^3^ Ph.D. Program for Biotechnology Industry, College of Life Sciences, China Medical University, Taichung, Taiwan; ^4^ Molecular Medicine Research Center, College of Medicine, Chang Gung University, Taoyuan, Taiwan

**Keywords:** single nucleotide polymorphism, Advanced HCC, arginine deprivation therapy, ASS1, HIF1A

## Abstract

**Background:**

Previous studies have identified three single nucleotide polymorphisms (SNPs): *GALNT14*-rs9679162, *WWOX*-rs13338697 and rs6025211. Their genotypes are associated with therapeutic outcomes in hepatocellular carcinoma (HCC). Herein, we examined whether these SNP genotypes could predict the clinical outcome of HCC patients treated with ADI-PEG 20.

**Methods:**

Totally 160 patients with advanced HCC, who had previously been enrolled in clinical trials, including 113 receiving ADI-PEG 20 monotherapy (cohort-1) and 47 receiving FOLFOX/ADI-PEG 20 combination treatment (cohort-2), were included retrospectively.

**Results:**

The *WWOX*-rs13338697-GG genotype was associated with favorable overall survival in cohort-1 patients (P = 0.025), whereas the rs6025211-TT genotype was associated with unfavorable time-to-tumor progression in cohort-1 (P = 0.021) and cohort-1 plus 2 patients (P = 0.008). As ADI-PEG 20 can reduce plasma arginine levels, we examined its pretreatment levels in relation to the *WWOX*-rs13338697 genotypes. Pretreatment plasma arginine levels were found to be significantly higher in patients carrying the *WWOX*-rs13338697-GG genotype (P = 0.006). We next examined the association of the *WWOX*-rs13338697 genotypes with WWOX tissue protein levels in 214 paired (cancerous/noncancerous) surgically resected HCC tissues (cohort-3). The *WWOX*-rs13338697-GG genotype was associated with decreased tissue levels of WWOX and ASS1. Mechanistic studies showed that WWOX and ASS1 levels were downregulated in hypoxic HCC cells. Silencing *WWOX* to mimic low WWOX protein expression in HCC in patients with the *WWOX*-rs13338697-GG genotype, enhanced HIF1A increment under hypoxia, further decreased ASS1, and increased cell susceptibility to ADI-PEG 20.

**Comclusion:**

In summary, the *WWOX*-rs13338697 and rs6025211 genotypes predicted treatment outcomes in ADI-PEG 20-treated advanced HCC patients. The *WWOX*-rs13338697-GG genotype was associated with lower tissue WWOX and ASS1 levels and higher pretreatment plasma arginine levels, resembling an arginine auxotrophic phenotype requires excessive extracellular arginine supply. Silencing *WWOX* to mimic HCC with the *WWOX*-rs13338697-GG genotype further stimulated HCC cell response to hypoxia through increased HIF1A expression, leading to further reduction of ASS1 and thus increased cell susceptibility to ADI-PEG 20.

## Introduction

In recent decades, cancer has become one of the leading causes of death in humans (1). Among the most common cancers, hepatocellular carcinoma (HCC) ranks the third leading cause of cancer-related death (2). Major risk factors for HCC include chronic hepatitis B or C infection and alcoholic liver diseases (3). Among all the therapeutic modalities for these patients, surgical removal of the liver tumor remains the most effective treatment (2). However, only a small number of HCC patients with acceptable liver function and in the early cancer stage without extrahepatic metastasis or large vessel invasion are qualified for surgical resection (4). In patients with unresectable HCC, the mainstay therapies include transcatheter arterial chemoembolization (TACE) using traditional or new embolization materials (for intermediate-stage HCC), oral targeted drugs, immunotherapy, radiotherapy, and chemotherapy (for advanced-stage HCC) (5). Despite the availability of these therapeutic treatments, a wide range of therapeutic responses has been observed in HCC patients, primarily due to the heterogeneity of their oncogenic pathways, resulting in significant changes in biological characteristics and genetic profiles (5, 6). Therefore, there is an urgent need for comprehensive therapeutic strategies to optimize responses to the therapies.

Depletion or reduction of exogenous amino acid sources using the amino acid-degrading enzymes has emerged as a new strategy to treat cancers possessing amino acid auxotroph phenotypes, such as arginine deprivation therapy (7–9). Two enzymatic drugs have been developed to achieve arginine deprivation in HCC patients: recombinant human arginase 1 (rhArg1) (10, 11) and pegylated arginine deiminase (ADI-PEG 20) (12, 13). Among these, the ADI-PEG 20 alone or in combination with other anticancer drugs has been demonstrated in several clinical trials as a safe and tolerable compound, including for advanced HCC (12, 14, 15). However, their efficacy in improving overall survival (OS), progression-free survival (PFS), time-to-tumor progression (TTP), and time-to-tumor response (TTR) remains unsatisfactory (12, 14–19). Hence, we sought an alternative approach to optimize the therapeutic efficacy of ADI-PEG 20 through re-evaluating the prognostic role of genetic markers in patients enrolled in clinical trials.

Previously, using a genome-wide association study (GWAS) followed by prospective validation, the single nucleotide polymorphism (SNP) genotypes of polypeptide N-acetyl-galactosaminyl-transferase 14 (*GALNT14*) have been identified as a predictor of response to chemotherapy in advanced HCC. Among them, a prominent SNP, rs9679162, was demonstrated to be associated with chemotherapeutic outcomes in HCC patients at Barcelona Clinic Liver Cancer (BCLC) stage C and TACE response in HCC patients at BCLC stage B. Subsequently, the same SNP has been identified as an outcome predictor for multiple gastrointestinal cancers, including colorectal cancer (for a review, see reference (20)). Furthermore, the *GALNT14* mutations have been associated with an increased risk of hereditary neuroblastoma (21).

In previous GWAS results, two other SNPs, *WWOX*-rs13338697 and rs6025211, have been shown to be assistive in predicting therapeutic responses in advanced HCC (22). Particularly, the *WWOX*-rs13338697 and rs6025211 genotypes have been associated with TTP and OS, respectively. Thus, a *post-hoc* analysis was conducted to re-evaluate the therapeutic efficacy of ADI-PEG 20 in advanced HCC patients enrolled in previous trials, in relationship to these SNPs.

## Materials and methods

### Patients enrolled in this study

Three cohorts were retrospectively enrolled in this study under the approval of the Institutional Review Board of Chang Gung Memorial Hospital in Taiwan [Approval number: 100-3119A1 (09 Nov 2011), 201601426A0 (02 Dec 2016), and 201900261B0 (04 Mar 2019)]. Patients enrolled in cohort-1 and -2 were diagnosed with advanced-stage HCC and had participated in clinical trials of ADI-PEG 20 (NCT01287585 and NCT02102022) in Taiwan. The cohort-1, containing 113 patients, received ADI-PEG 20 monotherapy, while cohort-2, including 47 patients, experienced ADI-PEG 20 plus modified FOLFOX6 (mFOLFOX6) chemotherapy (14, 15). In cohort-3, 214 HCC patients who had undergone surgical resection for HCC at Chang Gung Memorial Hospital and their paired liver tissue samples (cancerous/noncancerous) were included. Anonymous samples, including the blood samples of the cohort-1 and 2, and the resected tissues of the cohort-3, were retrospectively retrieved from the Polaris group (cohort-1 and 2) and the Tissue Bank of Chang Gung Memorial Hospital (cohort-3) for *post-hoc* analysis. Their de-linked clinical parameters, including age, gender, hepatitis virus infection, extrahepatic spread, macrovascular invasion, tumor number, largest tumor size (diameter), alpha-fetoprotein (AFP), albumin, bilirubin, creatinine, aspartate aminotransferase (AST), alanine aminotransferase (ALT), Child-Pugh score, albumin-bilirubin (ALBI) score, ALBI grade, and Eastern Cooperative Oncology Group (ECOG) performance status, were also retrieved. Specifically, de-linked clinical parameters for cohort-1 and 2 were obtained from the Polaris group, and those for cohort-3 were obtained retrospectively from electronic medical record review.

### Therapeutic outcomes evaluation

The duration of OS was calculated from the date on which the patients were included for randomization to the date of death, regardless of any causes, or the date of loss to follow-up. The objective tumor response was evaluated according to the Response Evaluation Criteria in Solid Tumors criteria (23). A >30% decrease in tumor volume, without progression in any target lesion or appearance of a new lesion, was defined as “response”. The duration of TTR was derived from the date on which the patients were included for randomization to the date of tumor response, which was censored at the date of leaving the trial. Progressive disease was defined as a >20% increase in the total tumor volume or appearance of a new lesion. The duration of TTP was calculated from the date of patient inclusion for randomization to that of disease progression, which was censored at the date of the last radiological assessment.

### SNP genotyping

SNP genotyping was conducted as previously described (22). Briefly, blood sample-derived genomic DNA was isolated using the QIAamp DNA mini kit (QIAGEN, Hilton, Germany, Cat: 51306) according to the instruction provided by the manufacturer. Semi-nested polymerase chain reaction (PCR) was conducted using the primers, Rs9679162-F1: 5’-TCACGAGGCCAACATTCTAG-3’; Rs9679162-R1: 5’-TTAGA-TTCTGCATGGCTCAC-3’; Rs9679162-R2: 5’-TCCCTCCTACTGAACCTCTCC-3’; Rs13338697-F1: 5’-ACTTCTGACAGCCATCCAGA-3’; Rs13338697-F2: 5’-ATCCTGCTAGCATGTTGACT-3’; Rs13338697-R2: 5’-ACTGTAGATGCCTTCCATCT-3’; Rs6025211-F1: 5’-ACATTCACAGAGAACTTGGC-3’; Rs6025211-R1: 5’-CAAGCAGTCCTTCCACCTTG-3’; Rs6025211-R2: 5’-AAAGTGCTGGGATTACAGG-T-3’. Amplified PCR products were gel-purified using the EasyPrep Gel & PCR Extraction Kit (BIOTOOLS, New Taipei City, Taiwan, Cat: TT-B14-3).

### Western blot analysis

Western blot analysis was performed as previously described (24). Briefly, the protein lysates were extracted by adding a sufficient amount of radio-immunoprecipitation assay (RIPA) buffer [20 mM Tris-HCl (pH 7.5), 150 mM NaCl, 1 mM Na_2_EDTA, 1 mM EGTA, and 1% NP-40] containing 1× protease inhibitor cocktail (BIOTOOLS, Cat: TAAR-BBI2) to the collected cells in tubes. The tubes were placed in a cold room at 4°C with continuous rotation for 30 min. The mixture was then centrifuged at 13,000 rpm for 15 min at 4°C. Protein concentration was assessed using a protein assay reagent (Bio-Rad, Hercules, CA, USA, Cat: 5000001). For each lane, 20 μg of denatured protein was loaded and separated on a 10% Bis-Tris-buffered polyacrylamide gel and transferred onto PVDF membranes for western blotting. The membranes were blocked with 5% milk before adding primary and secondary antibodies. Rabbit polyclonal antibodies against WW domain-containing oxidoreductase (WWOX) (Proteintech, Rosemont, IL, USA, Cat: 15299-1-AP; 1:1000 dilution), argininosuccinate synthase 1 (ASS1) (Proteintech, Cat: 16210-1-AP; 1:5000 dilution), hypoxia-inducible factor 1-alpha (HIF1A) (Proteintech, Cat: 20960-1-AP; 1:1000 dilution), and glyceraldehyde 3-phosphate dehydrogenase (GAPDH) (Proteintech, Cat: 10494-1-AP; 1:10000 dilution) were used in this study.

### Immunohistochemical staining

IHC was assayed as described previously (25). Briefly, after deparaffinization, rehydration, antigen retrieval, and endogenous peroxidase blockage, the tissue sections were hybridized with a specific primary antibody, followed by treatment with a horseradish peroxidase (HRP)-conjugated secondary antibody. The final signals were visualized by incubating the slides with the DAB substrate and counterstaining with hematoxylin solution. Rabbit polyclonal antibodies against WWOX (Proteintech, Cat: 15299-1-AP), and ASS1 (Proteintech, Cat: 16210-1-AP) were employed at a 1:100 dilution. Intensity of IHC images was acquired using ImageJ software (Fiji version).

### Cell culture and lentivirus-mediated silencing *WWOX*

HCC cell lines, Alexander [Research Resource Identifier (RRID): CVCL_0485], Huh7 (RRID: CVCL_0336), and HepG2 (RRID: CVCL_0027), were utilized in this study. All these cells were gifts from Dr. Kwang-Huei Lin at Chang Gung University, Taoyuan, Taiwan. These cells were maintained in Dulbecco’s Modified Eagle Medium (DMEM; for Huh7 cells) or alpha-MEM (for Alexander and HepG2 cells) with 10% fetal bovine serum in a humidified 37°C incubator with 5% CO_2_. Lentivirus-mediated gene silencing was conducted as previously described (26). An shRNA against LacZ was used. The WWOX shRNA clones, TRCN0000428967 and TRCN0000430007, were purchased from Academia Sinica, Taipei City, Taiwan.

### Cell viability assay

Cell viability assay was performed as previously mentioned (27). For the cell viability assay, 3×10^3^ cells were seeded for at least 16 h before adding ADI-PEG 20 to the culture medium. Alarmar Blue Cell Viability Reagent (Invitrogen, Waltham, MA, USA, Cat: DAL100) was used according to the instructions provided by manufacture.

### Statistical analysis

Dichotomized data are expressed as ratios (%) and compared using χ2 or Fisher’s exact test. Parametric data are expressed as mean ± standard deviation and compared using a two-sample *t*-test. Nonparametric or non-normally distributed data are expressed as median (range) and compared using the Mann–Whitney test. Univariate and multivariate Cox proportional hazards models were used to estimate survival for clinical and genotypic variables. After categorization, the Kaplan–Meier method was employed to estimate the survival probability between groups. The log-rank test was used to compare the survivals. A P < 0.05 was considered statistically significant. Statistical analyses were conducted using SPSS (IBM, Armonk, NY, USA, version 18.0) or Prism software (GraphPad Software, La Jolla, CA, USA, version 8.0).

## Results

### Baseline characteristics of ADI-PEG 20-treated patients included in this study

Two independent cohorts with a total of 160 patients with advanced-stage HCC were included in the *post-hoc* analysis. Among these, 113 and 47 who received ADI-PEG 20 monotherapy and ADI-PEG 20 plus mFOLFOX6 combination therapy were assigned to cohorts-1 and 2, respectively. **Table S1** summarizes and compares baseline clinical and genotypic characteristics of the two cohorts. Except for the distribution of tumor numbers, AST levels, and ECOG performance status, the differences in the remaining parameters, including the genotypic distribution of SNPs, were not statistically different. In cohort-1, patients had a higher number of tumors, a higher AST levels, and more patients with ECOG performance status “0” (P < 0.001, 0.003, and 0.002, respectively). However, the differences in tumor numbers and AST levels might originate from the inclusion criteria of the trials, in which patients with higher AST levels were excluded from the cohort-2 (14, 15).

### Clinicopathological factors and SNP genotypes are associated with OS, TTR, and TTP in advanced HCC patients receiving ADI-PEG 20 monotherapy

**Figure 1:**
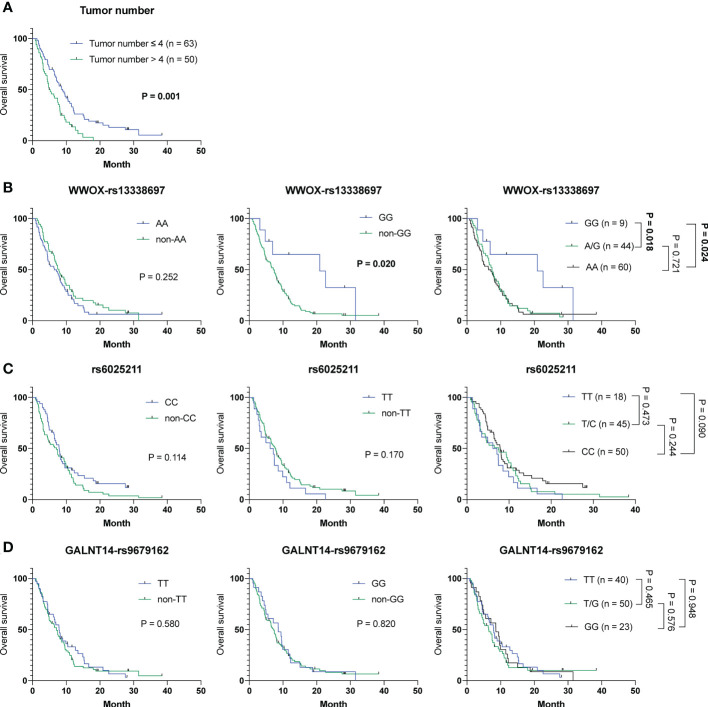
Analysis of clinicopathological factors and SNP genotypes in association with OS in HCC patients treated with ADI-PEG 20 monotherapy. Kaplan-Meier analysis for subgroups of patients with HCC stratified by **(A)** tumor number, **(B)** *WWOX*-rs13338697, **(C)** rs6025211, and **(D)** *GALNT14*-rs9679162 genotypes. P values were obtained by log-rank test, and < 0.05 was considered significant.

To investigate whether any of the parameters, including baseline characteristics and SNP genotypes, were correlated with OS in cohort-1, analyses using the Cox proportional hazards model were performed. Univariate analysis showed that patients with ≦ 4 tumors had a longer OS than those with > 4 tumors (mean OS, 10.6 months [95% CI: 8.5-12.7] versus 6.2 months [95% CI: 5.0-7.3]; P = 0.001). Additionally, patients with the *WWOX*-rs13338697-GG genotype had a longer OS compared to those with the *WWOX*-rs13338697-non-GG genotype (mean OS, 15.1 months [95% CI: 6.7-23.4] versus 8.1 months [95% CI: 6.8-9.3], P = 0.025) (**Table S2**). Furthermore, these two factors were independent predictors of OS in patients receiving ADI-PEG 20 monotherapy (adjusted P value = 0.003 and 0.045, respectively). The results of Kaplan-Meier analysis confirmed that tumor number and the *WWOX*-rs13338697-GG genotype could serve as independent factors to predict OS in cohort-1 (**Figures 1A, B** **)**. However, the rs6025211 and *GALNT14*-rs9679162 genotypes had no predictive value for OS (**Figures 1C, D** **)**.

Similar analyses were performed to understand whether these factors were associated with TTR and TTP in cohort-1. No correlation was observed between any of these parameters and TTR using the Cox proportional hazards model (**Table S3**) or Kaplan-Meier analysis (**Figure S1**).

**Figure 2:**
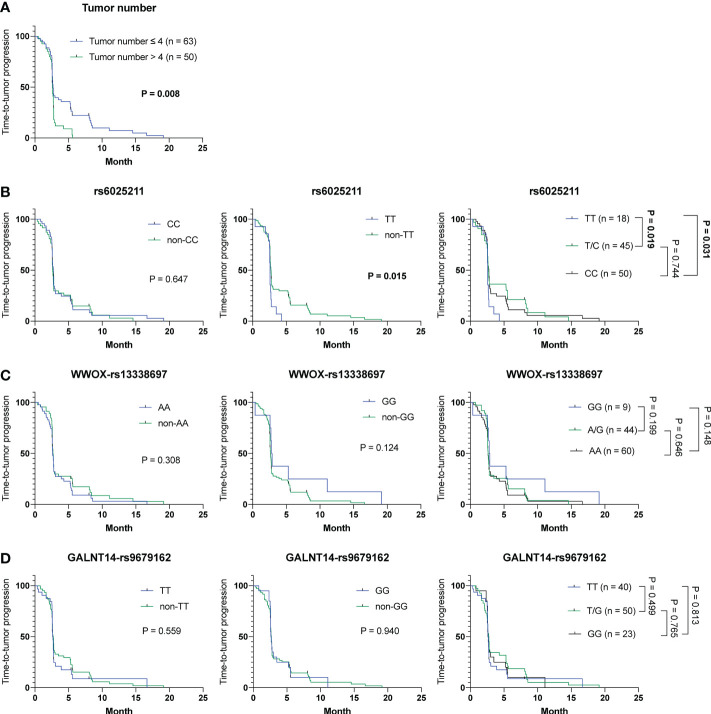
Analysis of clinicopathological factors and SNP genotypes in association with TTP in HCC patients treated with ADI-PEG 20 monotherapy. Kaplan-Meier analysis of subgroups of patients with HCC stratified by **(A)** tumor number, **(B)** rs6025211, **(C)** *WWOX*-rs13338697, and **(D)** *GALNT14*-rs9679162 genotypes. P values were obtained by log-rank test, and < 0.05 was considered significant.

Notably, extrahepatic spread, tumor number, and another factor, the rs6025211-TT genotype, were linked to TTP using univariate analysis in a Cox proportional hazards model (**Table S4**, P = 0.030, 0.011 and 0.021, respectively). Patients with extrahepatic spread were correlated with a shorter TTP compared with those without extrahepatic spread (mean TTP, 2.7 months [95% CI: 2.2-3.2] versus 4.5 months [95% CI: 2.5-6.4]). Also, patients with tumor number > 4 were associated with a shorter TTP compared with those with tumor number ≦ 4 (mean TTP, 2.1 months [95% CI: 1.7-2.5] versus 3.8 months [95% CI: 2.8-4.8]). For the rs6025211 genotypes, patients with the TT genotype had a shorter TTP than those with the non-TT genotype (mean TTP, 2.0 months [95% CI: 1.3-2.6] versus 3.3 months [95% CI: 2.6-4.0]). Multivariate analysis using a Cox proportional hazards model further demonstrated that only tumor number and the rs6025211-TT genotype were independent predictors of TTP in cohort-1 (P = 0.047 and 0.013, respectively). Kaplan-Meier analysis also revealed distinguishable stratification of patients by use of tumor number (P = 0.008) or the rs6025211-TT (P = 0.015 when compared to non-TT) in cohort-1 (**Figures 2A, B** **)**. No associations were found for the other SNP genotypes (**Figures 2C, D** **)**.

Collectively, the *WWOX*-rs13338697-GG and rs6025211-TT genotypes could predict the outcome of ADI-PEG 20 monotherapy independent of clinicopathological parameters, such as tumor number.

### Neither clinicopathological factors nor SNP genotypes are associated with clinical outcomes in patients receiving ADI-PEG 20 plus mFOLFOX6 combined therapy

To examine whether parameters including baseline characteristics and SNP genotypes correlated with OS, TTR and TTP in cohort-2, the aforementioned analyses were performed. However, no association was found between these parameters and prognoses was present, except for the Child-Pugh score (**Tables S5–7** and **Figures S2–4**). Unexpectedly, patients with a Child-Pugh score > 5 were associated with a shorter TTR (P = 0.039) (mean TTR, 2.1 months [95% CI: 0.0-8.2] versus 5.4 months [95% CI: 4.0-6.7]). However, only two patients had a Child-Pugh score > 5 (**Table S6**). Thus, there could be a bias.

### Clinicopathological factors and SNP genotypes are associated with OS, TTR and TTP in advanced HCC patients treated with ADI-PEG 20 monotherapy or combination therapy

**Figure 3:**
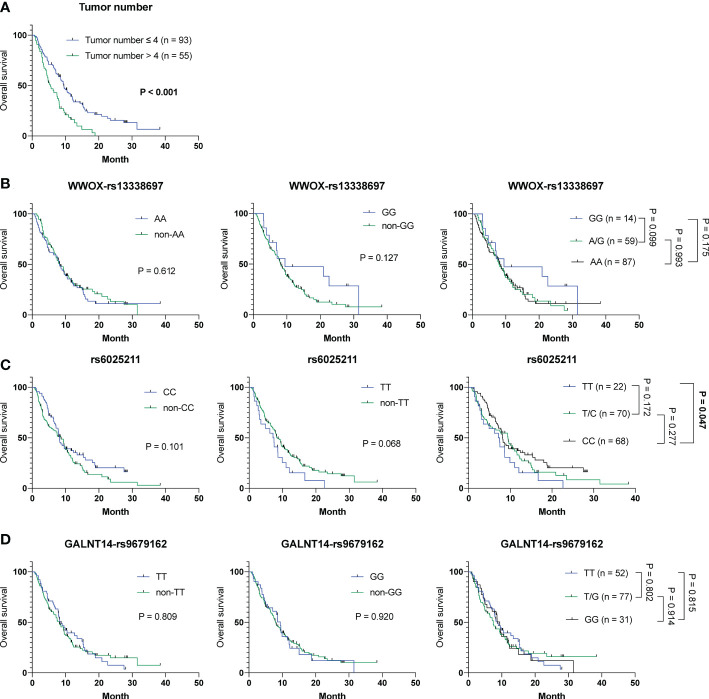
Analysis of clinicopathological factors and SNP genotypes in association with OS in all 160 HCC patients experiencing ADI-PEG 20 therapy. Kaplan-Meier analysis of subgroups of patients with HCC stratified by **(A)** tumor number, **(B)** *WWOX*-rs13338697, **(C)** rs6025211, and **(D)** *GALNT14*-rs9679162 genotypes. P values were obtained by log-rank test, and < 0.05 was considered significant.

**Figure 4:**
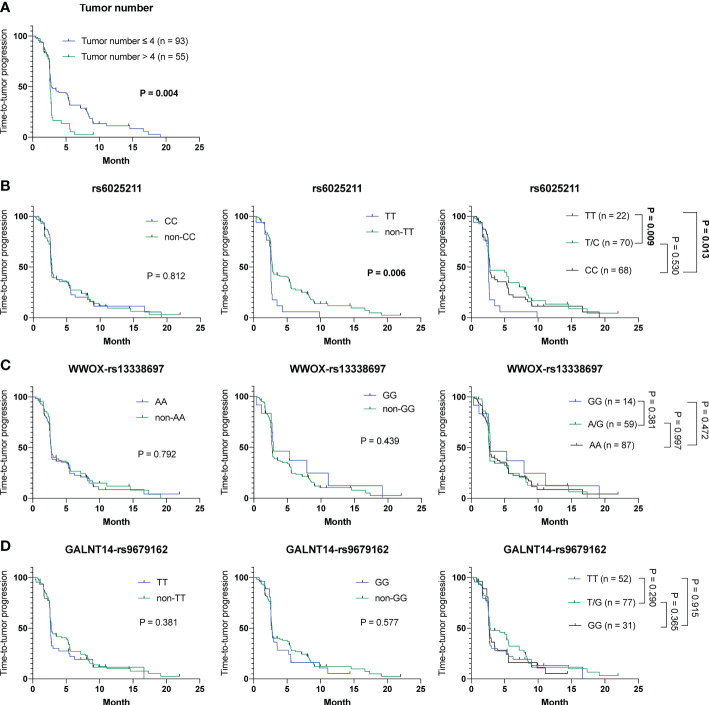
Analysis of clinicopathological factors and SNP genotypes in association with TTP in all 160 HCC patients experiencing ADI-PEG 20 therapy. Kaplan-Meier analysis of subgroups of patients with HCC stratified by **(A)** tumor number, **(B)** rs6025211, **(C)** *WWOX*-rs13338697, and **(D)** *GALNT14*-rs9679162 genotypes. P values were obtained by log-rank test, and < 0.05 was considered significant.

To understand whether parameters including baseline characteristics and SNP genotypes correlated with OS, TTR and TTP in all patients in cohort-1 and -2 combined, the aforementioned analyses were performed. The Cox proportional hazards model followed by Kaplan-Meier analysis demonstrated that only tumor number (**Table S8** and **Figure 3A**), but not other clinicopathological factors and SNP genotypes, served as an independent predictor for OS (**Figures 3B–D**). Except for ECOG performance status, where a higher status was associated with a shorter TTR (P = 0.024) (mean TTR, 3.8 months [95% CI: 3.0-4.6] versus 4.2 months [95% CI: 3.5-4.9]), no correlation was observed between the clinical/genetic parameters and TTR by using the Cox proportional hazards models (**Table S9**) and Kaplan-Meier analysis (**Figure S5**). However, extrahepatic spread (P = 0.030), tumor number > 4 (P = 0.005) and the rs6025211-TT genotype (P = 0.008) predicted unfavorable TTP, as analyzed by the univariate Cox proportional hazards model (**Table S10**). Multivariate analysis further showed that only tumor number (P = 0.047) and the rs6025211-TT genotype (P = 0.013) were independent predictors of TTP in all ADI-PEG 20-treated HCC patients. Kaplan-Meier analysis also confirmed this notion (P = 0.004 for comparison between patients stratified by tumor numbers, and P = 0.006 for comparison between subgroups of patients with the rs6025211-TT and non-TT genotypes) (**Figures 4A, B** **)**.

### The *WWOX*-rs13338697 genotype is not associated with clinical outcomes in HCC patients received surgical resection

**Figure 5:**
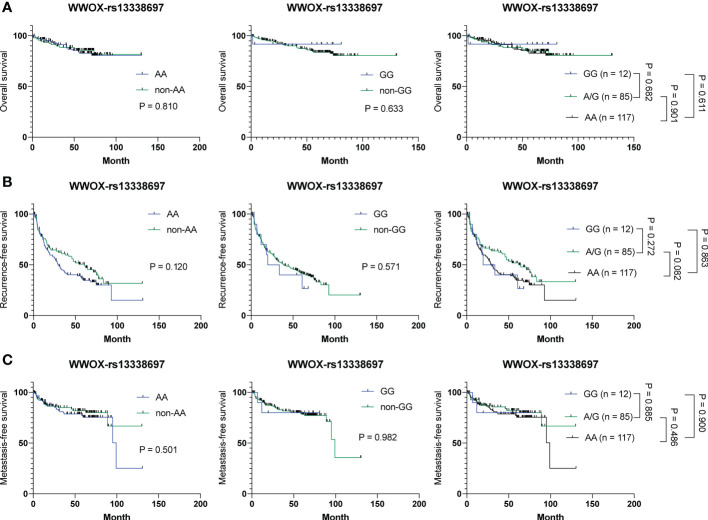
Analysis of the *WWOX*-rs13338697 genotypes in association with OS, RFS, and MFS in all 214 HCC patients receiving surgical resection. Kaplan-Meier analysis of **(A)** OS, **(B)** RFS, and **(C)** MFS of subgroups of patients with HCC stratified by the *WWOX*-rs13338697 genotypes. P values were obtained by log-rank test, and < 0.05 was considered significant.

To examine whether the *WWOX*-rs13338697 genotype is a prognostic factor but not a response predictor, tissue samples derived from 214 surgically resected HCC patients were retrieved for genotyping and prognosis analysis. As shown in **Figures 5A–C**, no correlation was observed between the *WWOX*-rs13338697 genotypes and the prognosis of patients with HCC in cohort-3, implying that this SNP predictor was specifically associated with the therapeutic efficacy of ADI-PEG 20.

### HCC patients with the *WWOX*-rs13338697-GG genotype have reduced tissue levels of WWOX and ASS1 and increased plasma arginine concentrations

**Figure 6:**
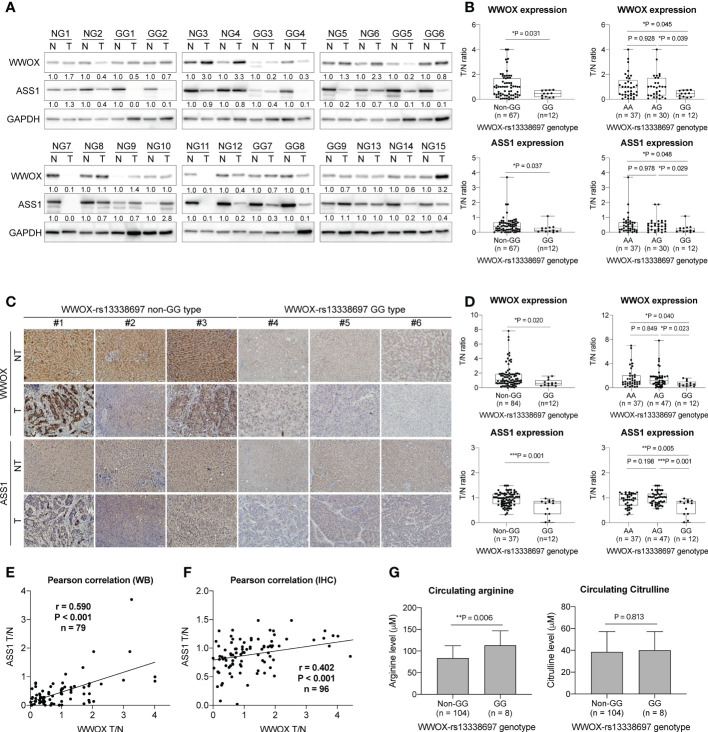
The *WWOX*-rs13338697 genotypes correlates with WWOX and ASS1 expression in HCC. **(A)** Western blot analysis of WWOX and ASS1 in patient-derived tissues. NG, Non-*WWOX*-rs13338697-GG genotype; GG, *WWOX*-rs13338697-GG genotype; N, Non-tumor; T, Tumor. Quantification results generated from **(A)** are summarized in **(B)**. T/N, Tumor/Nontumor ratio. **(C)** IHC analysis of WWOX and ASS1 in patient-derived sections. Quantification results generated from **(C)** are summarized in **(D)**. P values were obtained by two-tailed unpaired student *t*-test, and < 0.05 was considered significant. Pearson correlation of levels of ASS1 T/N and WWOX T/N generated using **(E)** Western blot analysis (WB) and **(F)** IHC are shown. **(G)** Arginine and Citrulline levels measured using plasma derived from cohort-1 patients. P values were obtained by two-tailed unpaired student *t*-test, and < 0.05 was considered significant. *P < 0.05; **P < 0.01; ***P < 0.001.

To further understand whether the *WWOX*-rs13338697 genotype affects the gene expression of *WWOX* and the expression of the critical enzyme ASS1 in HCC, western blot analysis and IHC staining were performed. As demonstrated in **Figures 6A, B**, the ratio of WWOX expression in tumorous/nontumorous (T/N) tissues was significantly lower in patients with the *WWOX*-rs13338697-GG genotype than in those with other genotypes (P = 0.031 compared to patients with the non-GG; P = 0.045 and 0.039 compared to patients with the AA and AG genotypes, respectively). Simultaneous examination of the T/N ratios of ASS1 levels further uncovered that it was also markedly decreased in patients with the *WWOX*-rs13338697-GG genotype compared to those with other genotypes (P = 0.037 compared to patients with the non-GG; P = 0.048 and 0.029 compared to patients with the AA and AG genotypes, respectively). IHC staining results also confirmed these findings that the *WWOX*-rs13338697-GG genotype correlated with a lower T/N ratios for WWOX (P = 0.020 compared to patients with the non-GG; P = 0.040 and 0.023 compared to patients with the AA and AG genotypes, respectively) and ASS1 (P = 0.001 compared to patients with the non-GG; P = 0.005 and 0.001 compared to patients with the AA and AG genotypes, respectively) in HCC (**Figures 6C, D** **)**. Pearson correlation analysis of western blotting and IHC staining results further demonstrated that the T/N ratios of WWOX and ASS1 were positively correlated (**Figures 6E, F** **)**.

To test whether the *WWOX*-rs13338697 genotypes correlates with plasma arginine or citrulline levels at baseline, plasma samples from cohort-1 patients were analyzed. Patients with the *WWOX*-rs13338697-GG genotype were associated with higher baseline (pretreatment) plasma arginine levels but not citrulline levels (P = 0.006) (**Figure 6G**).

### Silencing *WWOX* enhances HIF1A increase and ASS1 decrease in HCC cells in response to hypoxia

**Figure 7:**
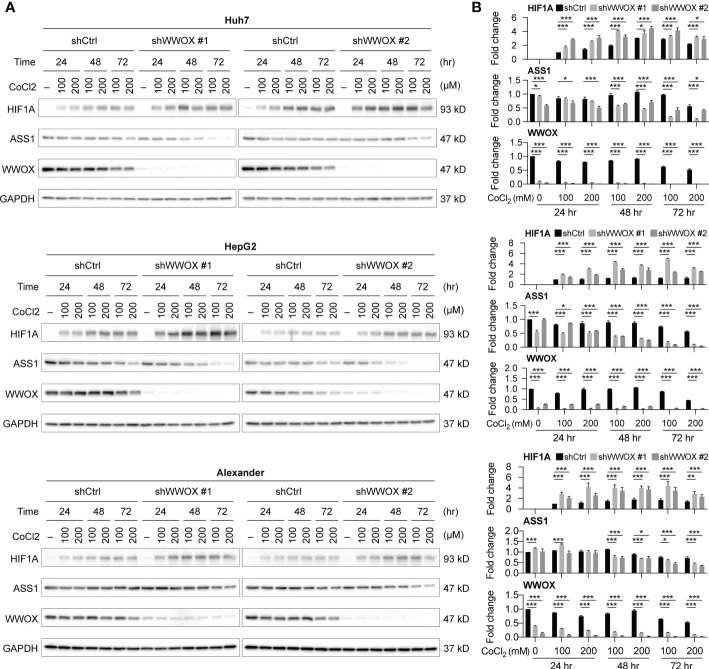
WWOX modulates HIF1A expression under hypoxia in HCC. **(A)** Western blot analysis of HIF1A, WWOX, and ASS1 expression in samples from HCC cells with the indicated treatments. Their quantitative results are summarized in **(B)**. P values were obtained by two-tailed paired student *t*-test, and < 0.05 was considered significant. *P < 0.05; **P < 0.01; ***P < 0.001.

To understand possible relationships between WWOX and ASS1 expression, a literature review was conducted. Notably, the effects of these molecules on hypoxia-mediated responses have been reported in several studies. In particular, Abu-Remaileh et al. showed that *WWOX* loss-of-function promotes HIF1A expression in HCC (28). Silberman et al. showed that increasing HIF1A suppressed ASS1 expression (29). Collectively, we hypothesize that lower levels of WWOX may suppress ASS1 expression through upregulating HIF1A in HCC cells. To test this hypothesis, HCC cells, with or without *WWOX* silencing, were treated with the hypoxia inducer, CoCl_2_, and harvested for western blot analysis. As shown in **Figures 7A, B**, HIF1A was undetectable without the addition of CoCl_2_. ASS1 levels remained similar in the three different HCC cell lines even after *WWOX* silencing (no statistical difference). However, a significant increase in HIF1A and a decrease in WWOX and ASS1 levels were noticed after CoCl_2_-induced hypoxia. These effects were further enhanced by *WWOX* silencing, particularly after 48 h of CoCl_2_ treatment. These findings indicate that silencing WWOX enhanced hypoxia-induced HIF1A expression, thereby repressing ASS1 expression in HCC cells.

### Silencing *WWOX* under hypoxic conditions enhances the susceptibility of HCC cells to ADI-PEG 20

**Figure 8:**
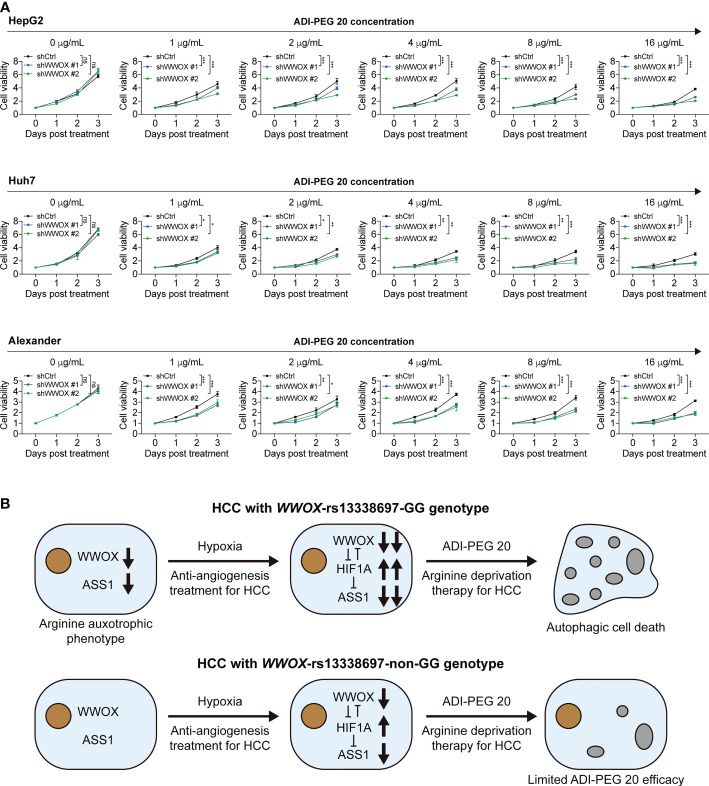
Silencing *WWOX* sensitizes HCC cells to ADI-PEG 20 under hypoxic conditions. **(A)** HCC cell viability when treated with the indicated concentrations of ADI-PEG 20, all under 100 μM CoCl_2_ treatment. P values were obtained by two-way ANOVA and < 0.05 was considered significant. *P < 0.05; **P < 0.01; ***P < 0.001. **(B)** The proposed working model of this study. HCC patients with the *WWOX*-rs13338697-GG genotype were associated with lower WWOX and ASS1 levels and higher plasma arginine levels. Decreased ASS1 and elevated extracellular arginine demand resemble the arginine auxotrophic phenotype. Low WWOX protein expression in HCC in patients with the *WWOX*-rs13338697-GG genotype enhances the cellular hypoxic response through an increase in HIF1A. Since HIF1A is a known repressor of ASS1, increasing HIF1A facilitates further decrease in ASS1. Subsequently, decreased in ASS1 increases the sensitivity of HCC cells to ADI-PEG 20. This model suggests a supportive role of hypoxia in ADI-PEG 20-based treatment. Gray bubbles indicate that autophagy has occurred. ns, no significance.

Although silencing WWOX indeed suppressed ASS1 expression in HCC cells, it remains unclear whether it alters the susceptibility of HCC cells to ADI-PEG 20. To answer this question, HCC cells, with or without *WWOX* silencing and CoCl_2_ treatment, were treated with different ADI-PEG 20 concentrations and then used for the cell viability assays. As shown in **Figure 8A**, growth curves of cells, with and without *WWOX* silencing, were not statistically different without the addition of ADI-PEG 20 (black, blue, and green lines for ADI-PEG 20 concentration at 0 μg/mL). Cells continued to grow under ADI-PEG 20 treatment. However, growth rates were attenuated (black lines for ADI-PEG 20 concentrations at 1, 2, 4, 8, and 16 μg/mL), suggesting that ADI-PEG 20 inhibited cell growth. Furthermore, ADI-PEG 20 had no or only weak cytotoxicity, and another combination agent was required to achieve better cytotoxic effects, as reported previously (30). Notably, silencing *WWOX* further attenuated cell growth when cells were treated with different concentrations of ADI-PEG 20 (blue and green lines for ADI-PEG 20 concentrations at 1, 2, 4, 8, and 16 μg/mL), indicating that reduced ASS1 expression after hypoxia induction and under WWOX knockdown enhanced the susceptibility of HCC cells to ADI-PEG 20.

## Discussion

To address the urgent need for new HCC therapeutic strategies, arginine deprivation therapy has been developed (e.g., ADI-PEG 20) in the past decades (12, 14, 15). However, unsatisfactory efficacies remained, with no significant difference in OS between groups of patients receiving placebo and ADI-PEG 20 treatment (15). However, ADI-PEG 20 prolonged OS for unknown reasons in a substantial proportion of enrolled patients. Herein, we discovered that the *WWOX*-rs13338697-GG genotype could predict the therapeutic efficacy of ADI-PEG 20 monotherapy in terms of prolonged OS (**Figure 1**). Additionally, another SNP, the rs6025211-TT genotype could predict unfavorable TTP in all ADI-PEG 20-treated HCC patients (**Figures 2**, **4**). These findings provide helpful information for patient selection in the upcoming ADI-PEG 20 clinical trials for HCC.

WWOX protein has been demonstrated to be a tumor suppressor in many types of cancers, including HCC, due to its downregulated expression and functional links. It modulates various oncogenic signaling pathways and alters the expression levels of several growth-related molecules, such as HIF1A, in either animal models or patient-derived tissues/cells (28, 31). Additionally, WWOX expression has been reported to be sensitive to (and suppressed under) hypoxia (32). In contrast, ASS1 expression has been demonstrated to be altered by hypoxic conditions, where elevated HIF1A binds directly to the *ASS1* promoter and leads to a significant decrease in ASS1 expression (29). Our results further confirmed these observations, as WWOX and ASS1 levels were markedly reduced under hypoxia (**Figure 7A**). Although the underlying molecular mechanisms by which the *WWOX*-rs13339697-GG genotype can lead to a lower WWOX expression remain unclear, there is a promising link between the *WWOX*-rs13339697-GG genotype and reduced WWOX expression. This correlation suggests that induction of hypoxia in HCC tissue during treatment with ADI-PEG 20 (for example, by TACE or TKI) may improve the therapeutic efficacy of ADI-PEG 20, and HCC patients harboring the *WWOX*-rs13339697-GG genotype should have further reduction of ASS1 levels and correspondingly further increase of tumor susceptibility to ADI-PEG 20 (**Figure 8B**).

A hypoxic microenvironment with elevated HIF1A that promotes cancer growth and creates anticancer drug resistance has been reported in patients treated with TACE and TKI treatments (33). However, we found that increased hypoxia in HCC may be more suitable for ADI-PEG 20 combination therapy. An increased hypoxic microenvironment correlates with higher HIF1A expression and represses the expression of ASS1, a key enzyme associated with therapeutic efficacy of ADI-PEG 20 in HCC, through transcriptional regulation of *ASS1* promoter activity (29). The results of this study suggest that either TACE or TKI may be an excellent combinatory therapy for ADI-PEG 20. This is because both treatments promote a hypoxic microenvironment (34), generating favorable conditions for ADI-PEG 20 targeting.

In surgically resected liver tissue, we found lower levels of ASS1 in patients with the *WWOX*-rs13339697-GG genotype (**Figures 6A** **–D**). This suggests a lower efficacy of *de novo* arginine biosynthesis, resembling the arginine auxotrophic phenotype. Therefore, tumors with the *WWOX*-rs13339697-GG genotype have an increased demand for extracellular arginine. Additionally, only patients with higher plasma arginine levels can maintain the progressive growth of ASS1-deficient HCC. This may be the reason why we found higher plasma arginine levels in cohort-1 patients with the *WWOX*-rs13339697-GG genotype (**Figure 6G**), as their tumor growth is highly dependent on exogenous arginine. Hence, ADI-PEG 20 suppressed the tumor growth drastically in patients with the *WWOX*-rs13339697-GG genotype, probably because these tumors displayed an arginine auxotrophic-like phenotype and were highly dependent on plasma arginine supply. In the future, it would be more compelling to use other advanced research methods to better understand the mechanisms by which the *WWOX*-rs13338697-GG genotype or the WWOX protein controls HCC growth and susceptibility to ADI-PEG 20. If tumor biopsies of patients with advanced HCC are available, more advanced tools can be used, for example, the systems biology approach using tumor growth-dependent prognostic subnetworks and the single-cell multi-omics analysis (35, 36).

Emerging evidence has suggested that immunotherapy, including using inhibitors to target programmed death-1 (PD-1) on effector T-cells or programmed death ligand-1 (PD-L1) on the tumor cell surface, providing adoptive cell therapy, and applying cancer vaccines, could be promising strategies to treat HCC (37, 38). According to the current understanding, the goal of achieving better efficacy of immunotherapy is to reduce immune-tolerance, activate full-function effector T-cell, and avoid expansion of regulatory T-cells (39, 40). Previously, certain amino acids and their metabolites have been documented to regulate the effectiveness of the immune system. Arginine and its metabolites, citrulline and nitric oxide (NO), can enhance effector T-cell proliferation and cytotoxicity (41); histidine and its metabolite, histamine, can promote lymphocyte growth and prevent apoptosis (42); and the metabolite of tryptophan from the kynurenine pathway, kynurenine, can result in immunosuppressive effects on T-cell and immune-tolerance to tumor cells (43). Notably, the deprivation of arginine by rhArg1, which leads to the production of urea and ornithine, or tryptophan by indoleamine 2,3-dioxygenase-1 (IDO1) or tryptophan 2,3-dioxygenase (TDO), which promotes the generation of the downstream metabolite kynurenine, has been demonstrated to induce immunosuppressive effects (44, 45). However, no immunosuppressive effects were observed when ADI-PEG 20 was used to achieve arginine deprivation either *in vitro*, *in vivo* (46), or in clinical settings (47). This may be because the product of ADI-PEG 20 treatment is citrulline, which, like arginine, has been documented to be able to promote T-cell proliferation (48). In this study, hypoxia enhanced the effectiveness of ADI-PEG 20 when WWOX expression was reduced (for example, patients with the *WWOX*-rs13338697-GG genotype). Since kynurenine biosynthesis is thought to be an oxygen-dependent event (49), it is reasonable to propose a combination therapy of ADI-PEG 20 and immunotherapy for the treatment of patients with advanced HCC, especially when a hypoxic tumor environment is achieved.

This study has limitations. For example, the limited availability of patient samples may bias the analysis of results. Furthermore, the relatively small number of cases, the rare frequency of the *WWOX*-rs13338697-GG genotype present in HCC patients, and the unelucidated molecular mechanisms behind the SNP genotype and gene expression all suggest the need for further investigation. Although these limitations exist, this study provides novel insights into the field of arginine deprivation therapy.

In conclusion, this study identified the *WWOX*-rs13338697-GG genotype as an independent predictor of OS in patients receiving ADI-PEG 20 monotherapy. Furthermore, the rs6025211-TT genotype was shown to be a predictor of TTP in cohort-1 (ADI-PEG 20 monotherapy) or in all patients experienced ADI-PEG 20 treatments (cohort-1 plus 2). HCC patients with the *WWOX*-rs13338697-GG genotype were associated with lower WWOX and ASS1 levels and higher plasma arginine levels. Decreased ASS1 and elevated extracellular arginine demand, resembling the arginine auxotrophic phenotype, are believed to be an important indicator of the efficacy of ADI-PEG 20. Cell-based assays demonstrated that silencing *WWOX* to mimic low WWOX protein expression in HCC in patients with the *WWOX*-rs13338697-GG genotype improved the cellular hypoxia response through a further increase of HIF1A. Since HIF1A is a known repressor of ASS1, increasing HIF1A leads to further decrease of ASS1. Subsequently, decrease in ASS1 increased the sensitivity of HCC cells to ADI-PEG 20.

## Data availability statement

The datasets presented in this study can be found in online repositories. The names of the repository/repositories and accession number(s) can be found in the article/**Supplementary Material**.

## Ethics statement

The studies involving human participants were reviewed and approved by IRB in Chang Gung Memorial Hospital, Taiwan and complied with the Declaration of Helsinki. The patients/participants provided their written informed consent to participate in this study. Written informed consent was obtained from the individual(s) for the publication of any potentially identifiable images or data included in this article.

## Author contributions

Conceptualization, Y-DC, C-HC, and C-TY. Methodology, C-TY. Formal analysis, Y-DC, H-FL, Y-CC, and C-HC. Original draft writing, Y-DC and C-HC. Manuscript review and editing, C-TY. Supervision, C-TY. Project administration, C-TY. Funding acquisition, C-TY. All authors have read and agreed to the published version of the manuscript.

## Funding

This research was funded by Polaris Pharmaceuticals, grant number SCRPD1L0171, and Chang Gung Memorial Hospital, Linkou branch, grant number CMRPG3M0891, to C-TY.

## Acknowledgments

The authors would like to thank all members of the Liver Research Center of Chang Gung Memorial Hospital, especially Ms. Yu-Ru Liang, and colleagues at Polaris Pharmaceuticals Inc.

## Conflict of interest

H-FL, Y-CC, C-HC were employed by Polaris Pharmaceuticals Inc. C-TY received funding from Polaris Pharmaceuticals Inc.

The remaining authors declare that the research was conducted in the absence of any commercial or financial relationships that could be construed as a potential conflict of interest.

## Publisher’s note

All claims expressed in this article are solely those of the authors and do not necessarily represent those of their affiliated organizations, or those of the publisher, the editors and the reviewers. Any product that may be evaluated in this article, or claim that may be made by its manufacturer, is not guaranteed or endorsed by the publisher.
