# Supplementary material for: WWOX-rs13338697 genotype predicts therapeutic efficacy of ADI-PEG 20 for patients with advanced hepatocellular carcinoma
Source: Front Oncol. 2022 Dec 2;12:996820. doi: 10.3389/fonc.2022.996820 (PMC9756969; doi:10.3389/fonc.2022.996820)
Supplement: Supplementary file 1 [file DataSheet_1.docx]

Supplementary Material

# Supplementary Figures and Tables

## Supplementary Figures

**
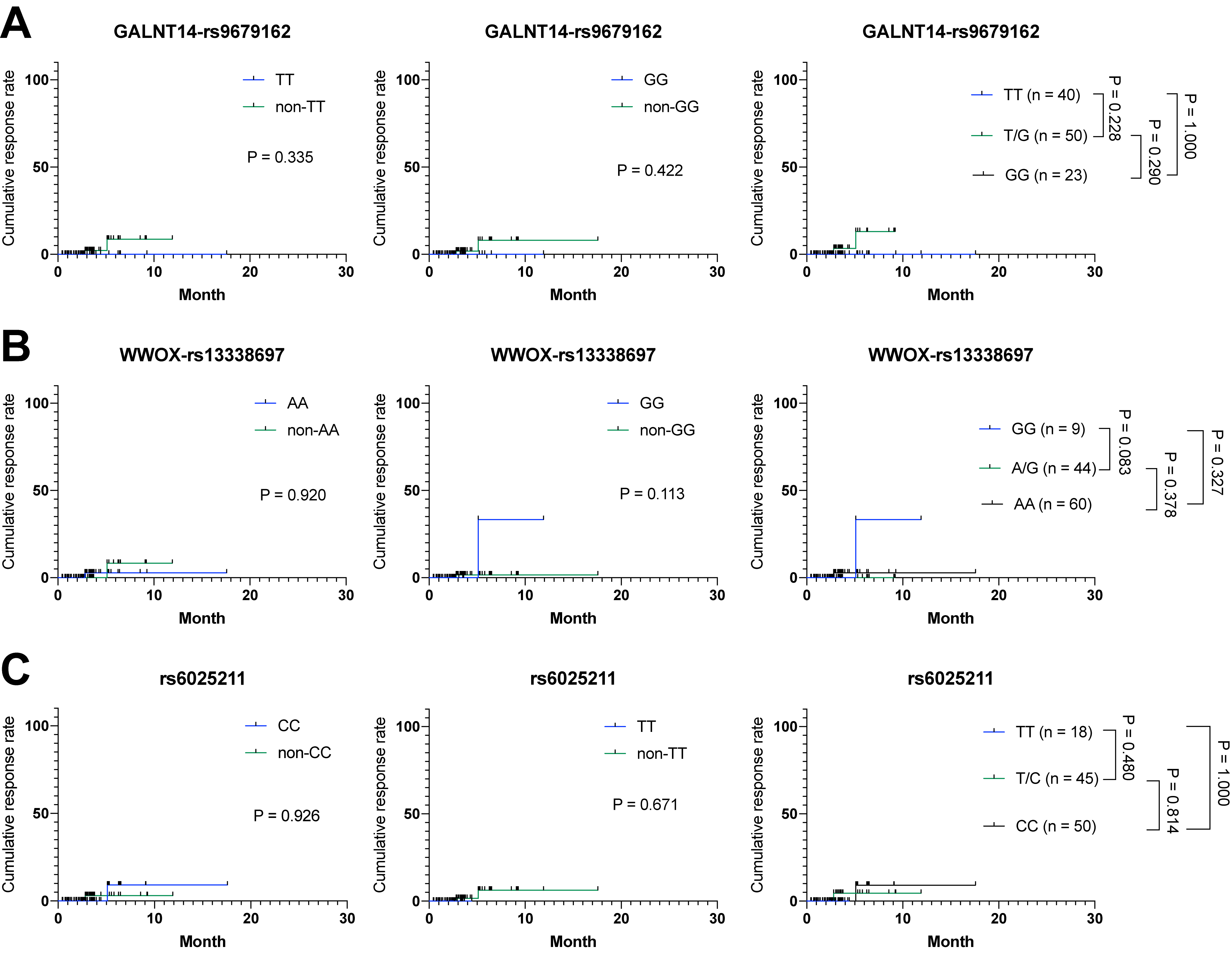
**

**Supplementary Figure 1.** Analysis of SNP genotypes in association with TTR in patients treated with ADI-PEG 20 monotherapy. Kaplan-Meyer analysis of subgroups of patients stratified by (A) *GALNT14*-rs9679162, (B) *WWOX*-rs13338697 and (C) rs6025211 genotypes. P values were obtained by log-rank test and < 0.05 was considered as significant.


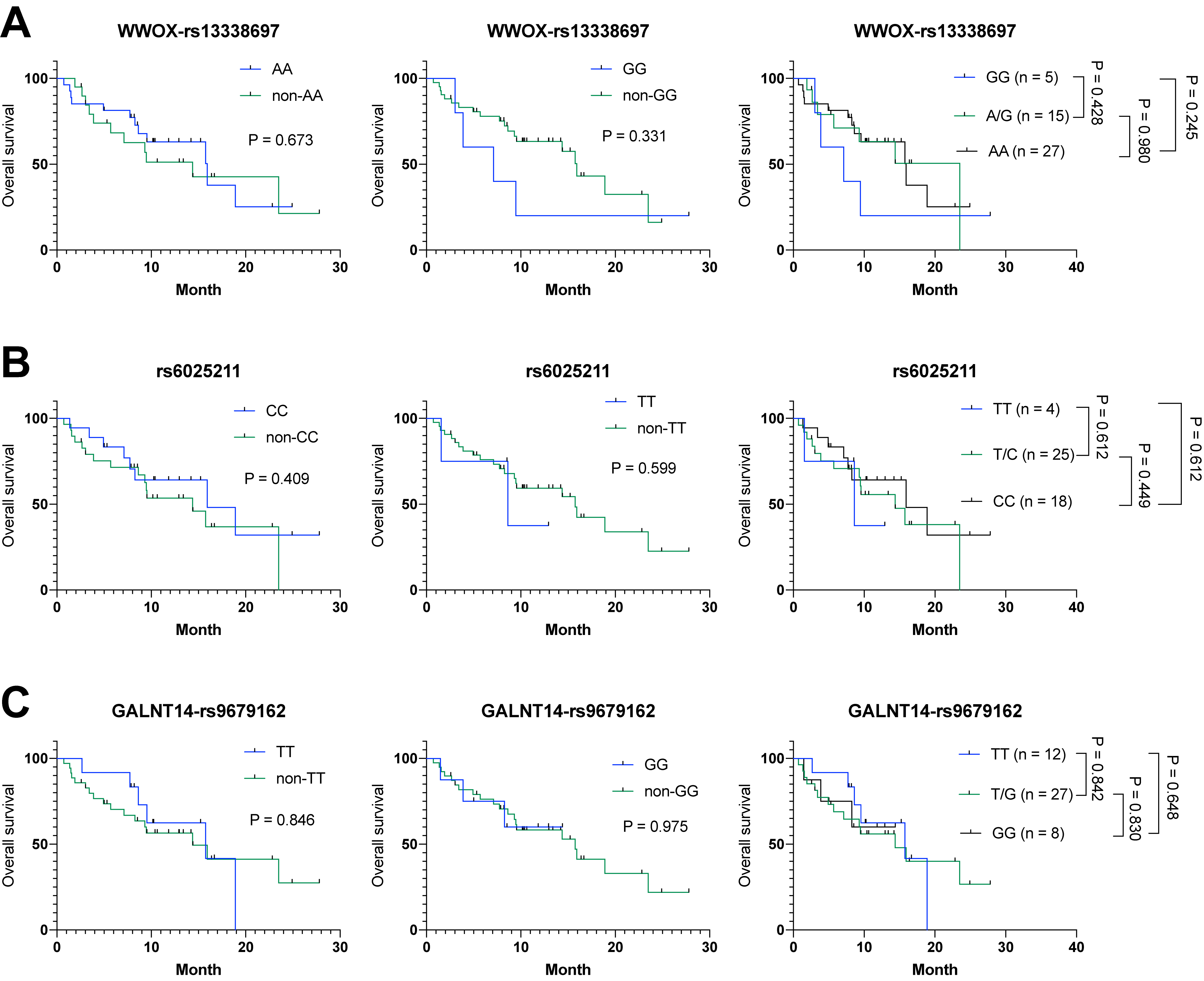


**Supplementary Figure 2.** Analysis of SNP genotypes in association with OS in patients with ADI-PEG 20 plus mFOLFOX6 combined therapy. Kaplan-Meyer analysis of subgroups of patients stratified by (A) *WWOX*-rs13338697, (B) rs6025211 and (C) *GALNT14*-rs9679162 genotypes. P values were obtained by log-rank test and < 0.05 was considered as significant.


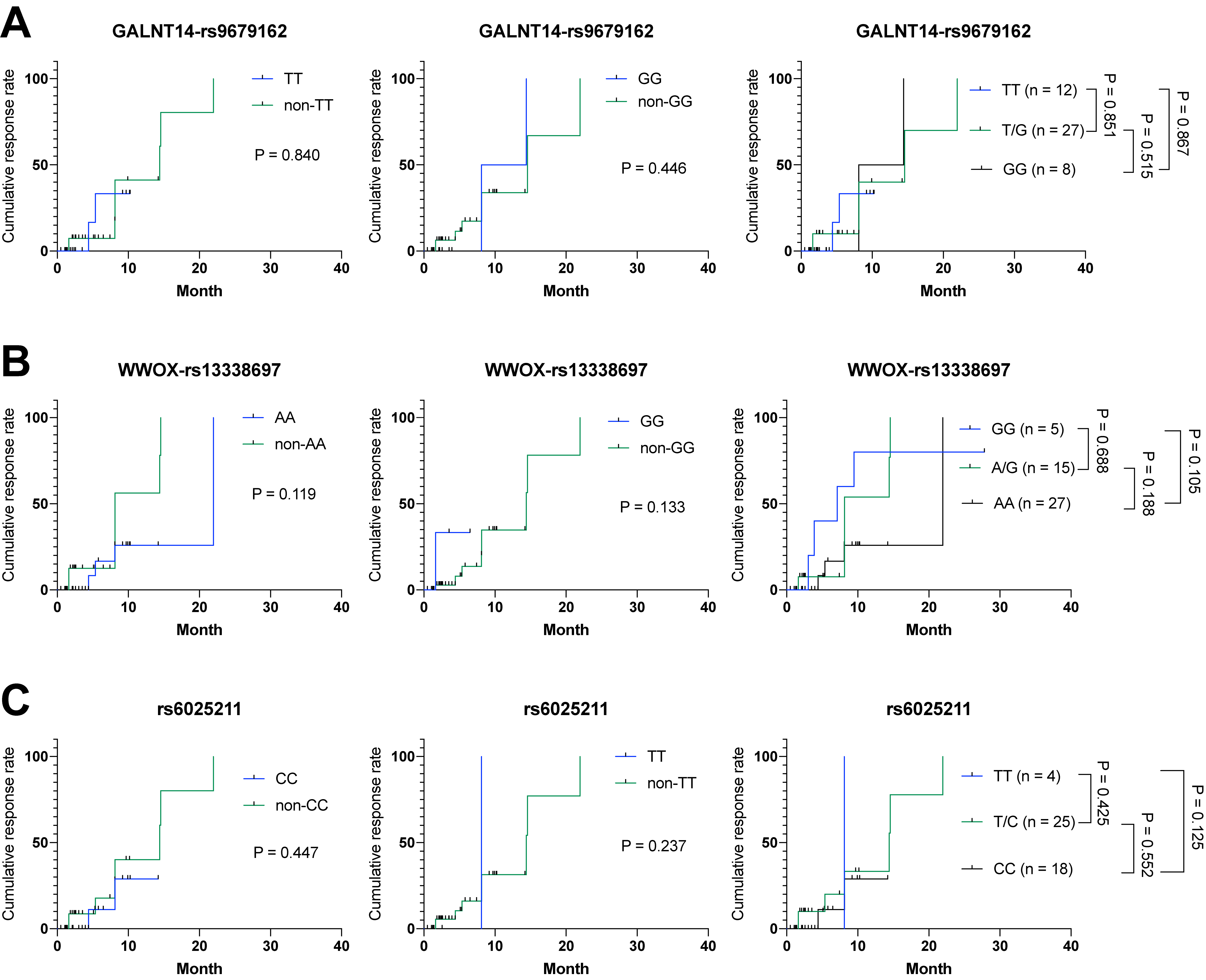


**Supplementary Figure 3.** Analysis of SNP genotypes in association with TTR in patients with ADI-PEG 20 plus mFOLFOX6 combined therapy. Kaplan-Meyer analysis of subgroups of patients stratified by (A) *GALNT14*-rs9679162, (B) *WWOX*-rs13338697 and (C) rs6025211 genotypes. P values were obtained by log-rank test and < 0.05 was considered as significant.


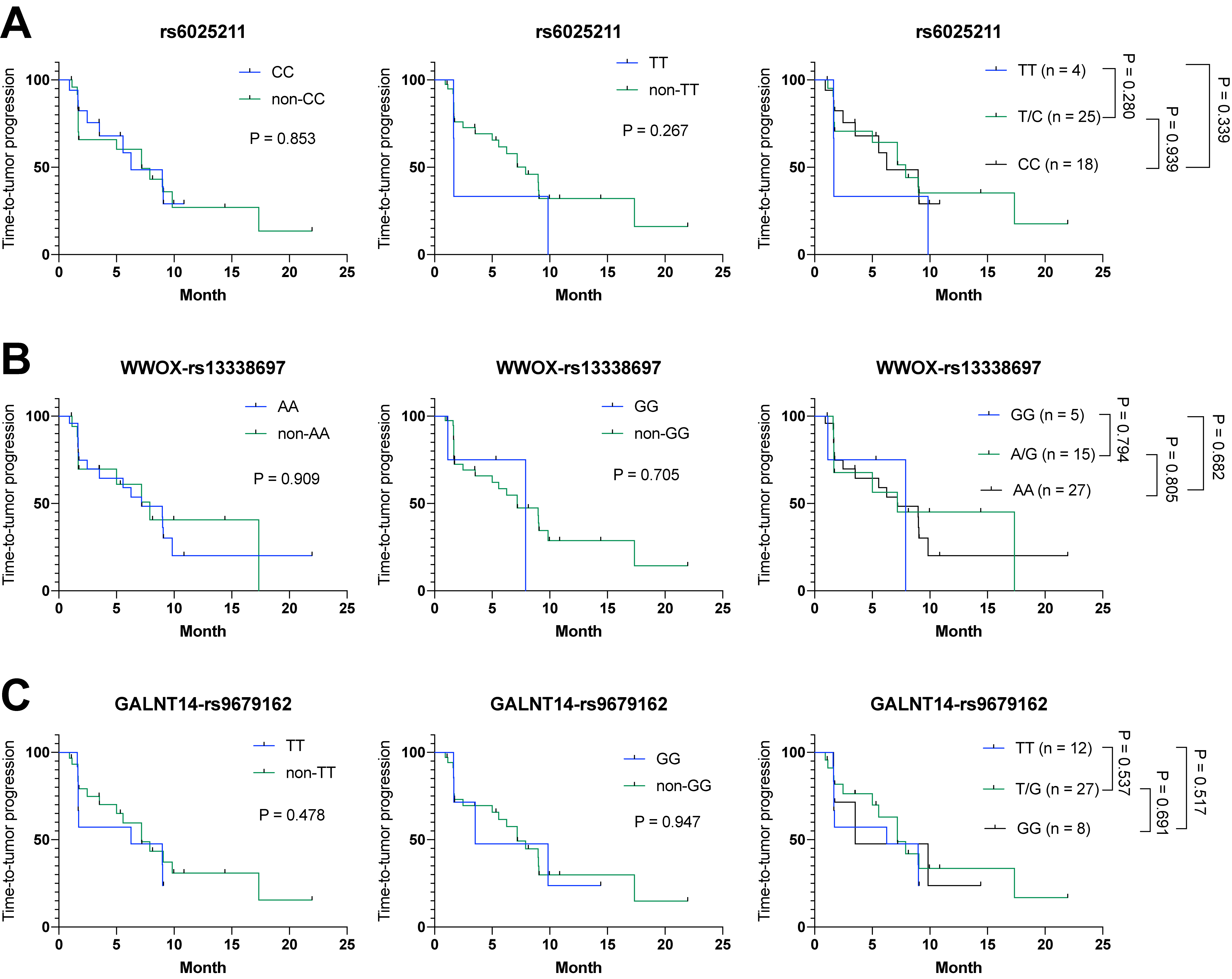


**Supplementary Figure 4.** Analysis of SNP genotypes in association with TTP in patients treated with ADI-PEG 20 plus mFOLFOX6 combination therapy. Kaplan-Meyer analysis of subgroups of patients stratified by (A) rs6025211, (B) *WWOX*-rs13338697 and (C) *GALNT14*-rs9679162 genotypes. P values were obtained by log-rank test and < 0.05 was considered as significant.


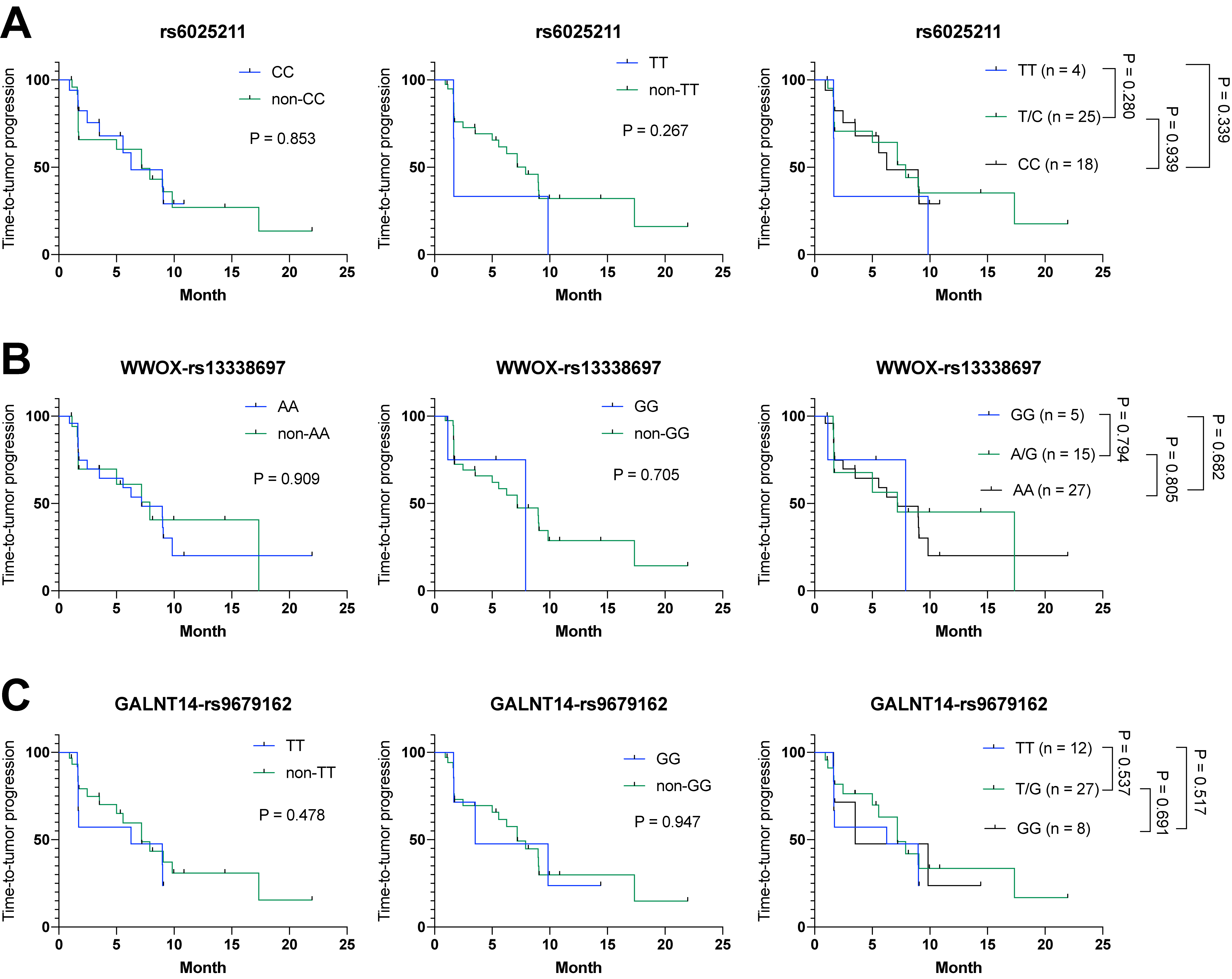


**Supplementary Figure 5.** Analysis of SNP genotypes in association with TTP in patients treated with ADI-PEG 20 plus mFOLFOX6 combination therapy. Kaplan-Meyer analysis of subgroups of patients stratified by (A) rs6025211, (B) *WWOX*-rs13338697 and (C) *GALNT14*-rs9679162 genotypes. P values were obtained by log-rank test and < 0.05 was considered as significant.

## Supplementary Tables

| **Table S1.** Baseline clinicopathological parameters and SNP genotypes between cohort-1 and -2. | | | | |
| --- | --- | --- | --- | --- |
| Clinical variables | Cohort-1 (n = 113) | Cohort-2 (n = 47) | P-value |  |
| Age, mean $\pm$ SD | 58.5 $\pm$ 11.5 | 58.4 $\pm$ 11.4 | 0.934 |  |
| Gender, male, n (%) | 96 (85.0%) | 41 (87.2%) | 0.708 |  |
| Anti-HBV, positive, n (%) | 80 (70.8%) | NA | NA |  |
| Anti-HCV, positive, n (%) | 29 (25.7%) | NA | NA |  |
| Tumor number, n (%)  < 3  ≥ 3 | 17 (15.0%)  96 (85.0%) | 27 (57.4%)  20 42.6%) | **< 0.001** |  |
| Largest tumor size, mm, mean $\pm$ SD | 58.6 $\pm$ 36.9 | 75.5 $\pm$ 50.9 | 0.090 |  |
| AFP, ng/mL, median (range) | 530.0 (1.8-1022847.2) | 632.2 (2.0-269000.0) | 0.514 |  |
| Albumin, g/dL, mean $\pm$ SD | 4.0 $\pm$ 0.5 | 4.1 $\pm$ 0.4 | 0.081 |  |
| Bilirubin, mg/dL, mean $\pm$ SD | 0.8 $\pm$ 0.4 | 0.9 $\pm$ 0.4 | 0.389 |  |
| Creatinine, mg/dL, mean $\pm$ SD | 0.8 $\pm$ 0.2 | 0.9 $\pm$ 0.2 | 0.076 |  |
| AST, U/L, mean $\pm$ SD | 68.3 $\pm$ 37.2 | 51.0 $\pm$ 28.9 | **0.003** |  |
| ALT, U/L, mean $\pm$ SD | 48.9 $\pm$ 31.8 | 40.6 $\pm$ 28.6 | 0.068 |  |
| Child-Pugh score, mean $\pm$ SD | 5.1 $\pm$ 0.5 | 5.0 $\pm$ 0.3 | 0.061 |  |
| ALBI score, mean $\pm$ SD | -2.7 $\pm$ 0.5 | -2.8 $\pm$ 0.4 | 0.204 |  |
| ALBI grade, 1/2, n (%) | 69 (61.1%) / 44 (38.9%) | 28 (59.6%) / 19 (40.4%) | 0.861 |  |
| ECOG performance status, 0/1, n (%) | 83 (73.5%) / 30 (26.5%) | 22 (46.8%) / 25 (53.2%) | **0.002** |  |
| *GALNT14*-rs9679162 “TT”, n (%) | 40 (35.4%) | 12 (25.5%) | 0.225 |  |
| *GALNT14*-rs9679162 “GG”, n (%) | 23 (20.4%) | 8 (17.0%) | 0.627 |  |
| *WWOX*-rs13338697 “AA”, n (%) | 60 (53.1%) | 27 (57.4%) | 0.615 |  |
| *WWOX*-rs13338697 “GG”, n (%) | 9 (8.0%) | 5 (10.6%) | 0.812 |  |
| rs6025211 “TT”, n (%) | 18 (15.9%) | 4 (8.5%) | 0.215 |  |
| rs6025211 “CC”, n (%) | 50 (44.2%) | 18 (38.3%) | 0.488 |  |

The bold indicates the significant P-value. P<0.05 was considered as significant. HBV, hepatitis B virus; HCV, hepatitis C virus; AFP, alpha-fetoprotein; AST, aspartate aminotransferase; ALT, alanine aminotransferase.

**Table S2.** Univariate and multivariate analysis of clinicopathological factors and SNP genotypes for OS in 113 ADI-PEG 20 monotherapy-treated advanced HCC patients.

|  |  | Univariate analysis | | | | | Multivariate analysis | | | | | |
| --- | --- | --- | --- | --- | --- | --- | --- | --- | --- | --- | --- | --- |
| Parameters | No. patients | Mean OS (95%CI) | HR (95% CI) | P |  | | | Adjusted HR (95% CI) | | P | |  |
| Age (years) |  |  |  |  | |  | | |  | |  |  |
| > 60.7 | 57 | 9.0 (7.1 – 11.0) |  |  | |  | | |  | |  |  |
| ≦ 60.7 | 56 | 8.3 (6.4 – 10.1) | 0.895 (0.603 – 1.328) | 0.581 | |  | | |  | |  |  |
| Gender |  |  |  |  | |  | | |  | |  |  |
| Male | 96 | 8.4 (7.1 – 9.7) |  |  | |  | | |  | |  |  |
| Female | 17 | 10.0 (4.8 – 15.1) | 1.101 (0.632 – 1.918) | 0.734 | |  | | |  | |  |  |
| Anti-HBV |  |  |  |  | |  | | |  | |  |  |
| Positive | 80 | 8.4 (6.9 – 9.9) |  |  | |  | | |  | |  |  |
| Negative | 33 | 9.3 (6.6 – 12.1) | 1.039 (0.675 – 1.602) | 0.861 | |  | | |  | |  |  |
| Anti-HCV |  |  |  |  | |  | | |  | |  |  |
| Positive | 29 | 9.6 (6.5 – 12.8) |  |  | |  | | |  | |  |  |
| Negative | 84 | 8.3 (6.9 – 9.8) | 0.804 (0.506 – 1.278) | 0.356 | |  | | |  | |  |  |
| Extrahepatic spread |  |  |  |  | |  | | |  | |  |  |
| Yes | 88 | 8.2 (6.9 – 9.6) |  |  | |  | | |  | |  |  |
| No | 25 | 10.3 (6.9 – 13.6) | 1.413 (0.862 – 2.316) | 0.170 | |  | | |  | |  |  |
| Macrovascular invasion |  |  |  |  | |  | | |  | |  |  |
| Yes | 41 | 7.1 (4.9 – 9.3) |  |  | |  | | |  | |  |  |
| No | 72 | 9.5 (7.9 – 11.2) | 1.459 (0.966 – 2.205) | 0.073 | |  | | |  | |  |  |
| Tumor number |  |  |  |  | |  | | |  | |  |  |
| > 4 | 50 | 6.2 (5.0 – 7.3) |  |  | |  | | |  | |  |  |
| ≦ 4 | 63 | 10.6 (8.5 – 12.7) | 1.983 (1.310 – 3.002) | **0.001** | | 1.885 (1.245 – 2.853) | | | **0.003** | |  |  |
| Largest tumor size (mm) |  |  |  |  | |  | | |  | |  |  |
| > 47.0 | 55 | 7.3 (5.9 – 8.8) |  |  | |  | | |  | |  |  |
| ≦ 47.0 | 58 | 9.9 (7.7 – 12.0) | 1.433 (0.962 – 2.135) | 0.077 | |  | | |  | |  |  |
| AFP (ng/mL) |  |  |  |  | |  | | |  | |  |  |
| > 400.0 | 61 | 7.6 (6.0 – 9.1) |  |  | |  | | |  | |  |  |
| ≦ 400.0 | 52 | 9.9 (7.8 – 12.1) | 1.398 (0.939 – 2.082) | 0.099 | |  | | |  | |  |  |
| Albumin (g/dL) |  |  |  |  | |  | | |  | |  |  |
| > 4.0 | 52 | 9.5 (7.5 – 11.6) |  |  | |  | | |  | |  |  |
| ≦ 4.0 | 61 | 7.9 (6.2 – 9.6) | 0.880 (0.594 – 1.305) | 0.526 | |  | | |  | |  |  |
| Bilirubin (mg/dL) |  |  |  |  | |  | | |  | |  |  |
| > 0.7 | 51 | 7.8 (5.8 – 9.8) |  |  | |  | | |  | |  |  |
| ≦ 0.7 | 62 | 9.4 (7.6 – 11.2) | 1.230 (0.829 – 1.825) | 0.304 | |  | | |  | |  |  |
| Creatinine (mg/dL) |  |  |  |  | |  | | |  | |  |  |
| > 0.8 | 62 | 8.7 (7.0 – 10.5) |  |  | |  | | |  | |  |  |
| ≦ 0.8 | 51 | 8.5 (6.5 – 10.6) | 1.021 (0.688 – 1.518) | 0.916 | |  | | |  | |  |  |
| AST (U/L) |  |  |  |  | |  | | |  | |  |  |
| > 61.0 | 56 | 7.6 (5.7 – 9.5) |  |  | |  | | |  | |  |  |
| ≦ 61.0 | 57 | 9.7 (7.8 – 11.5) | 1.387 (0.934 – 2.060) | 0.105 | |  | | |  | |  |  |
| ALT (U/L) |  |  |  |  | |  | | |  | |  |  |
| > 39.0 | 56 | 9.1 (7.1 – 11.0) |  |  | |  | | |  | |  |  |
| ≦ 39.0 | 57 | 8.3 (6.4 – 10.1) | 0.826 (0.557 – 1.225) | 0.342 | |  | | |  | |  |  |
| Child-Pugh score |  |  |  |  | |  | | |  | |  |  |
| > 5 | 19 | 7.4 (6.4 – 10.7) |  |  | |  | | |  | |  |  |
| ≦ 5 | 94 | 8.9 (7.4 – 10.4) | 1.011 (0.574 – 1.782) | 0.969 | |  | | |  | |  |  |
| ALBI score |  |  |  |  | |  | | |  | |  |  |
| > -2.7 | 60 | 9.6 (7.7 – 11.5) |  |  | |  | | |  | |  |  |
| ≦ -2.7 | 53 | 7.6 (5.7 – 9.4) | 1.300 (0.875 – 1.933) | 0.194 | |  | | |  | |  |  |
| ALBI grade |  |  |  |  | |  | | |  | |  |  |
| > 1 | 69 | 8.9 (7.3 – 10.7) |  |  | |  | | |  | |  |  |
| ≦ 1 | 44 | 8.1 (5.9 – 10.3) | 1.095 (0.730 – 1.641) | 0.662 | |  | | |  | |  |  |
| ECOG performance status |  |  |  |  | |  | | |  | |  |  |
| = 1 | 30 | 6.7 (5.2 – 8.3) |  |  | |  | | |  | |  |  |
| = 0 | 83 | 9.4 (7.7 – 11.1) | 1.509 (0.996 – 2.359) | 0.071 | |  | | |  | |  |  |
| *GALNT14*-rs9679162 “TT” |  |  |  |  | |  | | |  | |  |  |
| Yes | 40 | 9.0 (6.8 – 11.2) |  |  | |  | | |  | |  |  |
| No | 73 | 8.5 (6.8 – 10.1) | 0.890 (0.589 – 1.346) | 0.581 | |  | | |  | |  |  |
| *GALNT14*-rs9679162 “GG” |  |  |  |  | |  | | |  | |  |  |
| Yes | 23 | 9.4 (6.4 – 12.3) |  |  | |  | | |  | |  |  |
| No | 90 | 8.5 (7.0 – 10.0) | 0.946 (0.588 – 1.522) | 0.820 | |  | | |  | |  |  |
| *WWOX*-rs13338697 “AA” |  |  |  |  | |  | | |  | |  |  |
| Yes | 60 | 7.7 (6.0 – 9.4) |  |  | |  | | |  | |  |  |
| No | 53 | 9.7 (7.6 – 11.8) | 1.259 (0.848 – 1.869) | 0.254 | |  | | |  | |  |  |
| *WWOX*-rs13338697 “GG” |  |  |  |  | |  | | |  | |  |  |
| Yes | 9 | 15.1 (6.7 – 23.4) |  |  | |  | | |  | |  |  |
| No | 104 | 8.1 (6.8 – 9.3) | 0.382 (0.165 – 0.887) | **0.025** | | 0.420 (0.180 – 0.980) | | | **0.045** | |  |  |
| rs6025211 “TT” |  |  |  |  | |  | | |  | |  |  |
| Yes | 18 | 7.3 (4.5 – 10.1) |  |  | |  | | |  | |  |  |
| No | 95 | 8.9 (7.4 – 10.4) | 1.427 (0.855 – 2.382) | 0.173 | |  | | |  | |  |  |
| rs6025211 “CC” |  |  |  |  | |  | | |  | |  |  |
| Yes | 50 | 9.5 (7.5 – 11.6) |  |  | |  | | |  | |  |  |
| No | 63 | 8.0 (6.2 – 9.7) | 0.725 (0.485 – 1.083) | 0.116 | |  | | |  | |  |  |

Median value was used as cutoff for parametric data. The bold indicates the significant P-value. P<0.05 was considered as significant. OS, overall survival; HR, hazard ratio; CI, confidence interval; HBV, hepatitis B virus; HCV, hepatitis C virus; AFP, alpha-fetoprotein; AST, aspartate aminotransferase; ALT, alanine aminotransferase; ALBI, Albumin-bilirubin; ECOG, Eastern Cooperative Oncology Group.

**Table S3.** Univariate and multivariate analysis of clinicopathological factors and SNP genotypes for TTR in 113 ADI-PEG 20 monotherapy-treated advanced HCC patients.

|  |  | Univariate analysis | | | Multivariate analysis | | | | |
| --- | --- | --- | --- | --- | --- | --- | --- | --- | --- |
| Parameters | No. patients | Mean TTR (95%CI) | HR (95% CI) | P |  | Adjusted HR (95% CI) | | P |  |
| Age (years) |  |  |  |  |  | |  | | |
| > 60.7 | 57 | 3.9 (3.1 – 4.6) |  |  |  | |  | | |
| ≦ 60.7 | 56 | 3.3 (2.7 – 3.8) | 0.797 (0.050 – 12.752) | 0.872 |  | |  | | |
| Gender |  |  |  |  |  | |  | | |
| Male | 96 | 3.6 (3.1 – 4.1) |  |  |  | |  | | |
| Female | 17 | 3.5 (2.7 – 4.2) | 0.201 (0.012 – 3.242) | 0.258 |  | |  | | |
| Anti-HBV |  |  |  |  |  | |  | | |
| Positive | 80 | 3.4 (3.0 – 3.9) |  |  |  | |  | | |
| Negative | 33 | 4.0 (2.9 – 5.1) | 37.128 (0.000 – 10261440.2) | 0.572 |  | |  | | |
| Anti-HCV |  |  |  |  |  | |  | | |
| Positive | 29 | 4.6 (3.2 – 5.9) |  |  |  | |  | | |
| Negative | 84 | 3.2 (2.8 – 3.6) | 0.023 (0.000 – 3835.070) | 0.539 |  | |  | | |
| Extrahepatic spread |  |  |  |  |  | |  | | |
| Yes | 88 | 3.2 (2.8 – 3.6) |  |  |  | |  | | |
| No | 25 | 4.8 (3.3 – 6.3) | 0.519 (0.031 – 8.692) | 0.648 |  | |  | | |
| Macrovascular invasion |  |  |  |  |  | |  | | |
| Yes | 41 | 3.3 (2.4 – 4.2) |  |  |  | |  | | |
| No | 72 | 3.8 (3.2 – 4.3) | 2.788 (0.173 – 44.864) | 0.470 |  | |  | | |
| Tumor number |  |  |  |  |  | |  | | |
| > 4 | 50 | 2.9 (2.5 – 3.3) |  |  |  | |  | | |
| ≦ 4 | 63 | 4.1 (3.4 – 4.8) | 0.026 (0.000 – 11595.895) | 0.581 |  | |  | | |
| Largest tumor size (mm) |  |  |  |  |  | |  | | |
| > 47.0 | 55 | 3.2 (2.8 – 3.7) |  |  |  | |  | | |
| ≦ 47.0 | 58 | 3.9 (3.1 – 4.6) | 0.018 (0.000 – 1630.158) | 0.489 |  | |  | | |
| AFP (ng/mL) |  |  |  |  |  | |  | | |
| > 400.0 | 61 | 3.6 (2.9 – 4.3) |  |  |  | |  | | |
| ≦ 400.0 | 52 | 3.6 (3.0 – 4.1) | 0.015 (0.000 – 1271.968) | 0.466 |  | |  | | |
| Albumin (g/dL) |  |  |  |  |  | |  | | |
| > 4.0 | 52 | 3.7 (2.9 – 4.4) |  |  |  | |  | | |
| ≦ 4.0 | 61 | 3.5 (2.9 – 4.0) | 1.291 (0.081 – 20.647) | 0.857 |  | |  | | |
| Bilirubin (mg/dL) |  |  |  |  |  | |  | | |
| > 0.7 | 51 | 3.6 (2.9 – 4.2) |  |  |  | |  | | |
| ≦ 0.7 | 62 | 3.6 (3.0 – 4.2) | 0.996 (0.061 – 16.374) | 0.998 |  | |  | | |
| Creatinine (mg/dL) |  |  |  |  |  | |  | | |
| > 0.8 | 62 | 3.6 (3.0 – 4.2) |  |  |  | |  | | |
| ≦ 0.8 | 51 | 3.5 (2.8 – 4.2) | 0.702 (0.044 – 11.285) | 0.803 |  | |  | | |
| AST (U/L) |  |  |  |  |  | |  | | |
| > 61.0 | 56 | 3.6 (2.8 – 4.3) |  |  |  | |  | | |
| ≦ 61.0 | 57 | 3.6 (3.0 – 4.1) | 0.017 (0.000 – 1561.489) | 0.486 |  | |  | | |
| ALT (U/L) |  |  |  |  |  | |  | | |
| > 39.0 | 56 | 4.0 (3.2 – 4.7) |  |  |  | |  | | |
| ≦ 39.0 | 57 | 3.2 (2.8 – 3.7) | 0.008 (0.000 – 1337.483) | 0.431 |  | |  | | |
| Child-Pugh score |  |  |  |  |  | |  | | |
| > 5 | 19 | 3.4 (2.6 – 4.3) |  |  |  | |  | | |
| ≦ 5 | 94 | 3.6 (3.1 – 4.1) | 4.610 (0.288 – 73.868) | 0.280 |  | |  | | |
| ALBI score |  |  |  |  |  | |  | | |
| > -2.7 | 60 | 3.4 (2.8 – 4.0) |  |  |  | |  | | |
| ≦ -2.7 | 53 | 3.7 (3.1 – 4.4) | 1.140 (0.071 – 18.237) | 0.926 |  | |  | | |
| ALBI grade |  |  |  |  |  | |  | | |
| > 1 | 69 | 3.5 (2.8 – 4.2) |  |  |  | |  | | |
| ≦ 1 | 44 | 3.6 (3.0 – 4.2) | 1.315 (0.081 – 21.253) | 0.847 |  | |  | | |
| ECOG performance status |  |  |  |  |  | |  | | |
| = 1 | 30 | 3.0 (2.4 – 3.7) |  |  |  | |  | | |
| = 0 | 83 | 3.8 (3.2 – 4.3) | 0.033 (0.000 – 56791.910) | 0.643 |  | |  | | |
| *GALNT14*-rs9679162 “TT” |  |  |  |  |  | |  | | |
| Yes | 40 | 3.6 (2.6 – 4.5) |  |  |  | |  | | |
| No | 73 | 3.6 (3.1 – 4.1) | 0.027 (0.000 – 7443.874) | 0.572 |  | |  | | |
| *GALNT14*-rs9679162 “GG” |  |  |  |  |  | |  | | |
| Yes | 23 | 3.9 (2.9 – 4.8) |  |  |  | |  | | |
| No | 90 | 3.5 (3.0 – 4.0) | 0.032 (0.000 – 30056.970) | 0.624 |  | |  | | |
| *WWOX*-rs13338697 “AA” |  |  |  |  |  | |  | | |
| Yes | 60 | 3.4 (2.7 – 4.0) |  |  |  | |  | | |
| No | 53 | 3.8 (3.2 – 4.4) | 1.155 (0.072 – 18.594) | 0.919 |  | |  | | |
| *WWOX*-rs13338697 “GG” |  |  |  |  |  | |  | | |
| Yes | 9 | 4.5 (2.2 – 6.8) |  |  |  | |  | | |
| No | 104 | 3.5 (3.0 – 4.0) | 6.928 (0.430 – 111.562) | 0.172 |  | |  | | |
| rs6025211 “TT” |  |  |  |  |  | |  | | |
| Yes | 18 | 2.7 (2.2 – 3.3) |  |  |  | |  | | |
| No | 95 | 3.7 (3.2 – 4.3) | 0.039 (0.000 – 728491353) | 0.787 |  | |  | | |
| rs6025211 “CC” |  |  |  |  |  | |  | | |
| Yes | 50 | 4.0 (3.3 – 4.7) |  |  |  | |  | | |
| No | 63 | 3.2 (2.7 – 3.8) | 0.877 (0.055 – 14.028) | 0.926 |  | |  | | |

Median value was used as cutoff for parametric data. The bold indicates the significant P-value. P<0.05 was considered as significant. OS, overall survival; HR, hazard ratio; CI, confidence interval; HBV, hepatitis B virus; HCV, hepatitis C virus; AFP, alpha-fetoprotein; AST, aspartate aminotransferase; ALT, alanine aminotransferase; ALBI, Albumin-bilirubin; ECOG, Eastern Cooperative Oncology Group.

**Table S4.** Univariate and multivariate analysis of clinicopathological factors and SNP genotypes for TTP in 113 ADI-PEG 20 monotherapy-treated advanced HCC patients.

|  |  | Univariate analysis | | | Multivariate analysis | | | | |
| --- | --- | --- | --- | --- | --- | --- | --- | --- | --- |
| Parameters | No. patients | Mean TTP (95%CI) | HR (95% CI) | P |  | Adjusted HR (95% CI) | | P |  |
| Age (years) |  |  |  |  |  | |  | | |
| > 60.7 | 57 | 3.4 (2.6 – 4.3) |  |  |  | |  | | |
| ≦ 60.7 | 56 | 2.7 (1.9 – 3.5) | 0.854 (0.558 – 1.308) | 0.467 |  | |  | | |
| Gender |  |  |  |  |  | |  | | |
| Male | 96 | 3.1 (2.4 – 3.8) |  |  |  | |  | | |
| Female | 17 | 3.1 (2.0 – 4.2) | 0.888 (0.507 – 1.557) | 0.679 |  | |  | | |
| Anti-HBV |  |  |  |  |  | |  | | |
| Positive | 80 | 3.0 (2.3 – 3.7) |  |  |  | |  | | |
| Negative | 33 | 3.2 (2.1 – 4.3) | 0.963 (0.613 – 1.514) | 0.870 |  | |  | | |
| Anti-HCV |  |  |  |  |  | |  | | |
| Positive | 29 | 3.8 (2.5 – 5.2) |  |  |  | |  | | |
| Negative | 84 | 2.8 (2.2 – 3.5) | 0.855 (0.535 – 1.366) | 0.512 |  | |  | | |
| Extrahepatic spread |  |  |  |  |  | |  | | |
| Yes | 88 | 2.7 (2.2 – 3.2) |  |  |  | |  | | |
| No | 25 | 4.5 (2.5 – 6.4) | 1.814 (1.059 – 3.105) | **0.030** | 1.513 (0.850 – 2.692) | | 0.159 | | |
| Macrovascular invasion |  |  |  |  |  | |  | | |
| Yes | 41 | 3.1 (1.7 – 4.4) |  |  |  | |  | | |
| No | 72 | 3.1 (2.5 – 3.6) | 1.020 (0.637 – 1.634) | 0.934 |  | |  | | |
| Tumor number |  |  |  |  |  | |  | | |
| > 4 | 50 | 2.1 (1.7 – 2.5) |  |  |  | |  | | |
| ≦ 4 | 63 | 3.8 (2.8 – 4.8) | 1.819 (1.149 – 2.878) | **0.011** | 1.639 (1.007 – 2.667) | | **0.047** | | |
| Largest tumor size (mm) |  |  |  |  |  | |  | | |
| > 47.0 | 55 | 2.8 (2.2 – 3.5) |  |  |  | |  | | |
| ≦ 47.0 | 58 | 3.3 (2.3 – 4.3) | 1.253 (0.813 – 1.931) | 0.307 |  | |  | | |
| AFP (ng/mL) |  |  |  |  |  | |  | | |
| > 400.0 | 61 | 2.8 (2.1 – 3.5) |  |  |  | |  | | |
| ≦ 400.0 | 52 | 3.5 (2.5 – 4.4) | 1.347 (0.896 – 2.106) | 0.145 |  | |  | | |
| Albumin (g/dL) |  |  |  |  |  | |  | | |
| > 4.0 | 52 | 3.3 (2.3 – 4.2) |  |  |  | |  | | |
| ≦ 4.0 | 61 | 2.9 (2.1 – 3.7) | 1.070 (0.699 – 1.637) | 0.757 |  | |  | | |
| Bilirubin (mg/dL) |  |  |  |  |  | |  | | |
| > 0.7 | 51 | 2.9 (2.0 – 3.9) |  |  |  | |  | | |
| ≦ 0.7 | 62 | 3.2 (2.5 – 3.9) | 0.787 (0.507 – 1.222) | 0.286 |  | |  | | |
| Creatinine (mg/dL) |  |  |  |  |  | |  | | |
| > 0.8 | 62 | 3.3 (2.4 – 4.1) |  |  |  | |  | | |
| ≦ 0.8 | 51 | 2.9 (2.1 – 3.6) | 0.784 (0.513 – 1.200) | 0.262 |  | |  | | |
| AST (U/L) |  |  |  |  |  | |  | | |
| > 61.0 | 56 | 3.1 (2.2 – 3.9) |  |  |  | |  | | |
| ≦ 61.0 | 57 | 3.1 (2.3 – 3.9) | 1.104 (0.721 – 1.689) | 0.649 |  | |  | | |
| ALT (U/L) |  |  |  |  |  | |  | | |
| > 39.0 | 56 | 3.3 (2.4 – 4.2) |  |  |  | |  | | |
| ≦ 39.0 | 57 | 2.8 (2.1 – 3.6) | 0.777 (0.507 – 1.192) | 0.248 |  | |  | | |
| Child-Pugh score |  |  |  |  |  | |  | | |
| > 5 | 19 | 3.5 (1.3 – 5.6) |  |  |  | |  | | |
| ≦ 5 | 94 | 3.0 (2.4 – 3.6) | 0.746 (0.411 – 1.353) | 0.335 |  | |  | | |
| ALBI score |  |  |  |  |  | |  | | |
| > -2.7 | 60 | 2.8 (1.9 – 3.7) |  |  |  | |  | | |
| ≦ -2.7 | 53 | 3.3 (2.5 – 4.1) | 0.884 (0.575 – 1.360) | 0.575 |  | |  | | |
| ALBI grade |  |  |  |  |  | |  | | |
| > 1 | 69 | 3.0 (1.9 – 4.0) |  |  |  | |  | | |
| ≦ 1 | 44 | 3.1 (2.4 – 3.9) | 0.821 (0.526 – 1.281) | 0.386 |  | |  | | |
| ECOG performance status |  |  |  |  |  | |  | | |
| = 1 | 30 | 2.3 (1.5 – 3.0) |  |  |  | |  | | |
| = 0 | 83 | 3.4 (2.6 – 4.1) | 1.360 (0.834 – 2.219) | 0.218 |  | |  | | |
| *GALNT14*-rs9679162 “TT” |  |  |  |  |  | |  | | |
| Yes | 40 | 2.7 (1.7 – 3.6) |  |  |  | |  | | |
| No | 73 | 3.3 (2.5 – 4.1) | 1.140 (0.725 – 1.794) | 0.570 |  | |  | | |
| *GALNT14*-rs9679162 “GG” |  |  |  |  |  | |  | | |
| Yes | 23 | 3.1 (2.1 – 4.1) |  |  |  | |  | | |
| No | 90 | 3.1 (2.4 – 3.8) | 1.019 (0.611 – 1.702) | 0.941 |  | |  | | |
| *WWOX*-rs13338697 “AA” |  |  |  |  |  | |  | | |
| Yes | 60 | 2.7 (2.0 – 3.4) |  |  |  | |  | | |
| No | 53 | 3.5 (2.5 – 4.5) | 1.240 (0.810 – 1.897) | 0.322 |  | |  | | |
| *WWOX*-rs13338697 “GG” |  |  |  |  |  | |  | | |
| Yes | 9 | 5.2 (0.5 – 9.9) |  |  |  | |  | | |
| No | 104 | 2.9 (2.4 – 3.4) | 0.551 (0.250 – 1.213) | 0.139 |  | |  | | |
| rs6025211 “TT” |  |  |  |  |  | |  | | |
| Yes | 18 | 2.0 (1.3 – 2.6) |  |  |  | |  | | |
| No | 95 | 3.3 (2.6 – 4.0) | 2.016 (1.114 – 3.650) | **0.021** | 2.119 (1.171 – 3.837) | | **0.013** | | |
| rs6025211 “CC” |  |  |  |  |  | |  | | |
| Yes | 50 | 3.5 (2.5 – 4.5) |  |  |  | |  | | |
| No | 63 | 2.8 (2.0 – 3.5) | 0.907 (0.592 – 1.392) | 0.656 |  | |  | | |

Median value was used as cutoff for parametric data. The bold indicates the significant P-value. P<0.05 was considered as significant. OS, overall survival; HR, hazard ratio; CI, confidence interval; HBV, hepatitis B virus; HCV, hepatitis C virus; AFP, alpha-fetoprotein; AST, aspartate aminotransferase; ALT, alanine aminotransferase; ALBI, Albumin-bilirubin; ECOG, Eastern Cooperative Oncology Group.

**Table S5.** Univariate and multivariate analysis of clinicopathological factors and SNP genotypes for OS in 47 ADI-PEG 20 and FOLFOX combination therapy-treated advanced HCC patients.

|  |  | Univariate analysis | | | | Multivariate analysis | | | | | |  |
| --- | --- | --- | --- | --- | --- | --- | --- | --- | --- | --- | --- | --- |
| Parameters | No. patients | Mean OS (95%CI) | HR (95% CI) | P |  | | | Adjusted HR (95% CI) | | P |  |  |
| Age (years) |  |  |  |  | | |  | |  | | | |
| > 60.3 | 23 | 9.3 (6.7 – 11.9) |  |  | | |  | |  | | | |
| ≦ 60.3 | 24 | 10.8 (7.8 – 13.9) | 1.048 (0.461 – 2.381) | 0.911 | | |  | |  | | | |
| Gender |  |  |  |  | | |  | |  | | | |
| Male | 41 | 10.0 (7.8 – 12.1) |  |  | | |  | |  | | | |
| Female | 6 | 11.0 (6.8 – 15.2) | 1.533 (0.355 – 6.614) | 0.567 | | |  | |  | | | |
| Tumor number |  |  |  |  | | |  | |  | | | |
| > 3 | 13 | 9.9 (6.7 – 13.1) |  |  | | |  | |  | | | |
| ≦ 3 | 22 | 10.3 (6.9 – 13.7) | 1.762 (0.713 – 4.354) | 0.220 | | |  | |  | | | |
| Largest tumor size (mm) |  |  |  |  | | |  | |  | | | |
| > 59.5 | 17 | 8.9 (5.5 – 12.2) |  |  | | |  | |  | | | |
| ≦ 59.5 | 17 | 11.5 (7.8 – 15.1) | 1.242 (0.514 – 3.000) | 0.631 | | |  | |  | | | |
| AFP (ng/mL) |  |  |  |  | | |  | |  | | | |
| > 400.0 | 23 | 8.0 (6.2 – 9.9) |  |  | | |  | |  | | | |
| ≦ 400.0 | 16 | 10.4 (7.0 – 13.7) | 1.632 (0.591 – 4.505) | 0.345 | | |  | |  | | | |
| Albumin (g/dL) |  |  |  |  | | |  | |  | | | |
| > 4.1 | 22 | 9.3 (6.8 – 11.8) |  |  | | |  | |  | | | |
| ≦ 4.1 | 25 | 10.8 (7.7 – 13.9) | 1.049 (0.451 – 2.438) | 0.911 | | |  | |  | | | |
| Bilirubin (mg/dL) |  |  |  |  | | |  | |  | | | |
| > 0.8 | 21 | 9.2 (6.4 – 12.1) |  |  | | |  | |  | | | |
| ≦ 0.8 | 26 | 10.8 (8.0 – 13.6) | 1.773 (0.776 – 4.050) | 0.174 | | |  | |  | | | |
| Creatinine (mg/dL) |  |  |  |  | | |  | |  | | | |
| > 0.9 | 24 | 9.0 (6.5 – 11.5) |  |  | | |  | |  | | | |
| ≦ 0.9 | 23 | 11.2 (8.1 – 14.3) | 1.850 (0.790 – 4.329) | 0.156 | | |  | |  | | | |
| AST (U/L) |  |  |  |  | | |  | |  | | | |
| > 43.5 | 24 | 10.6 (7.8 – 13.5) |  |  | | |  | |  | | | |
| ≦ 43.5 | 23 | 9.5 (6.7 – 12.4) | 1.128 (0.493 – 2.579) | 0.776 | | |  | |  | | | |
| ALT (U/L) |  |  |  |  | | |  | |  | | | |
| > 32.0 | 24 | 10.7 (7.9 – 13.4) |  |  | | |  | |  | | | |
| ≦ 32.0 | 23 | 9.4 (6.5 – 12.4) | 0.932 (0.410 – 2.121) | 0.868 | | |  | |  | | | |
| Child-Pugh score |  |  |  |  | | |  | |  | | | |
| > 5 | 2 | 4.1 (0.0 – 24.0) |  |  | | |  | |  | | | |
| ≦ 5 | 45 | 10.4 (8.4 – 12.4) | 2.781 (0.354 – 21.859) | 0.331 | | |  | |  | | | |
| ALBI score |  |  |  |  | | |  | |  | | | |
| > -2.8 | 27 | 10.8 (7.8 – 13.8) |  |  | | |  | |  | | | |
| ≦ -2.8 | 20 | 9.2 (6.7 – 11.6) | 0.921 (0.389 – 2.181) | 0.851 | | |  | |  | | | |
| ALBI grade |  |  |  |  | | |  | |  | | | |
| > 1 | 19 | 10.3 (6.2 – 14.3) |  |  | | |  | |  | | | |
| ≦ 1 | 28 | 10.0 (7.9 – 12.0) | 1.262 (0.542 – 2.938) | 0.590 | | |  | |  | | | |
| ECOG performance status |  |  |  |  | | |  | |  | | | |
| = 1 | 25 | 10.6 (7.8 – 13.3) |  |  | | |  | |  | | | |
| = 0 | 22 | 9.5 (6.6 – 12.5) | 0.521 (0.225 – 1.209) | 0.129 | | |  | |  | | | |
| *GALNT14*-rs9679162 “TT” |  |  |  |  | | |  | |  | | | |
| Yes | 12 | 11.0 (8.0 – 13.9) |  |  | | |  | |  | | | |
| No | 35 | 9.8 (7.3 – 12.3) | 0.911 (0.356 – 2.334) | 0.846 | | |  | |  | | | |
| *GALNT14*-rs9679162 “GG” |  |  |  |  | | |  | |  | | | |
| Yes | 8 | 8.3 (4.4 – 12.1) |  |  | | |  | |  | | | |
| No | 39 | 10.5 (8.2 – 12.7) | 1.020 (0.296 – 3.512) | 0.975 | | |  | |  | | | |
| *WWOX*-rs13338697 “AA” |  |  |  |  | | |  | |  | | | |
| Yes | 27 | 10.0 (7.5 – 12.5) |  |  | | |  | |  | | | |
| No | 20 | 10.2 (6.8 – 13.6) | 0.837 (0.367 – 1.909) | 0.673 | | |  | |  | | | |
| *WWOX*-rs13338697 “GG” |  |  |  |  | | |  | |  | | | |
| Yes | 5 | 10.3 (0.0 – 22.8) |  |  | | |  | |  | | | |
| No | 42 | 10.1 (8.1 – 12.0) | 1.716 (0.571 – 5.153) | 0.336 | | |  | |  | | | |
| rs6025211 “TT” |  |  |  |  | | |  | |  | | | |
| Yes | 4 | 7.9 (0.4 – 15.4) |  |  | | |  | |  | | | |
| No | 43 | 10.3 (8.2 – 12.4) | 1.481 (0.339 – 6.473) | 0.602 | | |  | |  | | | |
| rs6025211 “CC” |  |  |  |  | | |  | |  | | | |
| Yes | 18 | 11.4 (7.8 – 15.0) |  |  | | |  | |  | | | |
| No | 29 | 9.3 (6.9 – 11.6) | 0.696 (0.292 – 1.655) | 0.412 | | |  | |  | | | |

Median value was used as cutoff for parametric data. The bold indicates the significant P-value. P<0.05 was considered as significant. OS, overall survival; HR, hazard ratio; CI, confidence interval; HBV, hepatitis B virus; HCV, hepatitis C virus; AFP, alpha-fetoprotein; AST, aspartate aminotransferase; ALT, alanine aminotransferase; ALBI, Albumin-bilirubin; ECOG, Eastern Cooperative Oncology Group.

**Table S6.** Univariate and multivariate analysis of clinicopathological factors and SNP genotypes for TTR in 47 ADI-PEG 20 and FOLFOX combination therapy-treated advanced HCC patients.

|  |  | Univariate analysis | | | | | Multivariate analysis | | | | | | | | |  |  |  |  |
| --- | --- | --- | --- | --- | --- | --- | --- | --- | --- | --- | --- | --- | --- | --- | --- | --- | --- | --- | --- |
| Parameters | No. patients | Mean TTR (95%CI) | HR (95% CI) | P | |  | | | | | Adjusted HR (95% CI) | | | | P | | | |  |
| Age (years) |  |  |  |  | | | |  | | | |  | | | | |  |  |  |
| > 60.3 | 23 | 4.6 (3.0 – 6.2) |  |  | | | |  | | | |  | | | | |  |  |  |
| ≦ 60.3 | 24 | 5.9 (3.7 – 8.1) | 2.222 (0.525 – 9.406) | 0.278 | | | |  | | | |  | | | | |  |  |  |
| Gender |  |  |  |  | | | |  | | | |  | | | | |  |  |  |
| Male | 41 | 4.9 (3.5 – 6.4) |  |  | | | |  | | | |  | | | | |  |  |  |
| Female | 6 | 7.5 (2.9 – 12.0) | 0.896 (0.178 – 4.521) | 0.895 | | | |  | | | |  | | | | |  |  |  |
| Tumor number |  |  |  |  | | | |  | | | |  | | | | |  |  |  |
| > 3 | 13 | 4.4 (2.6 – 6.2) |  |  | | | |  | | | |  | | | | |  |  |  |
| ≦ 3 | 22 | 5.3 (3.4 – 7.2) | 5.298 (0.545 – 51.457) | 0.151 | | | |  | | | |  | | | | |  |  |  |
| Largest tumor size (mm) |  |  |  |  | | | |  | | | |  | | | | |  |  |  |
| > 59.5 | 17 | 4.5 (3.0 – 5.9) |  |  | | | |  | | | |  | | | | |  |  |  |
| ≦ 59.5 | 17 | 5.3 (2.9 – 7.7) | 3.339 (0.342 – 32.570) | 0.300 | | | |  | | | |  | | | | |  |  |  |
| AFP (ng/mL) |  |  |  |  | | | |  | | | |  | | | | |  |  |  |
| > 400.0 | 23 | 4.1 (2.9 – 5.2) |  |  | | | |  | | | |  | | | | |  |  |  |
| ≦ 400.0 | 16 | 6.8 (3.5 – 10.1) | 6.328 (0.719 – 55.735) | 0.096 | | | |  | | | |  | | | | |  |  |  |
| Albumin (g/dL) |  |  |  |  | | | |  | | | |  | | | | |  |  |  |
| > 4.1 | 22 | 5.2 (3.1 – 7.2) |  |  | | | |  | | | |  | | | | |  |  |  |
| ≦ 4.1 | 25 | 5.3 (3.4 – 7.2) | 0.654 (0.161 – 2.647) | 0.551 | | | |  | | | |  | | | | |  |  |  |
| Bilirubin (mg/dL) |  |  |  |  | | | |  | | | |  | | | | |  |  |  |
| > 0.8 | 21 | 4.1 (2.3 – 6.0) |  |  | | | |  | | | |  | | | | |  |  |  |
| ≦ 0.8 | 26 | 6.2 (4.2 – 8.1) | 2.224 (0.571 – 8.668) | 0.249 | | | |  | | | |  | | | | |  |  |  |
| Creatinine (mg/dL) |  |  |  |  | | | |  | | | |  | | | | |  |  |  |
| > 0.9 | 24 | 4.3 (2.8 – 5.7) |  |  | | | |  | | | |  | | | | |  |  |  |
| ≦ 0.9 | 23 | 6.3 (4.0 – 8.6) | 0.552 (0.109 – 2.805) | 0.474 | | | |  | | | |  | | | | |  |  |  |
| AST (U/L) |  |  |  |  | | | |  | | | |  | | | | |  |  |  |
| > 43.5 | 24 | 5.3 (3.5 – 7.1) |  |  | | | |  | | | |  | | | | |  |  |  |
| ≦ 43.5 | 23 | 5.2 (3.1 – 7.3) | 1.109 (0.295 – 4.163) | 0.879 | | | |  | | | |  | | | | |  |  |  |
| ALT (U/L) |  |  |  |  | | | |  | | | |  | | | | |  |  |  |
| > 32.0 | 24 | 5.0 (3.3 – 6.7) |  |  | | | |  | | | |  | | | | |  |  |  |
| ≦ 32.0 | 23 | 5.5 (3.3 – 7.7) | 0.879 (0.234 – 3.3013) | 0.848 | | | |  | | | |  | | | | |  |  |  |
| Child-Pugh score |  |  |  | |  | | | | |  | | | |  | | | | | |
| > 5 | 2 | 2.1 (0.0 – 8.2) |  |  | | | |  | | | |  | | | | |  |  |  |
| ≦ 5 | 45 | 5.4 (4.0 – 6.7) | 18.500 (1.157 – 295.769) | **0.039** | | | |  | | | |  | | | | |  |  |  |
| ALBI score |  |  |  | |  | | | | |  | | | |  | | | | | |
| > -2.8 | 27 | 5.0 (3.2 – 6.8) |  |  | | | |  | | | |  | | | | |  |  |  |
| ≦ -2.8 | 20 | 5.6 (3.4 – 7.8) | 2.704 (0.557 – 13.138) | 0.217 | | | |  | | | |  | | | | |  |  |  |
| ALBI grade |  |  |  |  | | | | |  | | | |  | | | | |  |  |
| > 1 | 19 | 4.7 (2.3 – 7.1) |  |  | | | |  | | | |  | | | | |  |  |  |
| ≦ 1 | 28 | 5.6 (4.0 – 7.2) | 1.172 (0.302 – 4.547) | 0.818 | | | |  | | | |  | | | | |  |  |  |
| ECOG performance status |  |  |  | |  | | | | |  | | | |  | | | | | |
| = 1 | 25 | 4.7 (3.2 – 6.2) |  |  | | | |  | | | |  | | | | |  |  |  |
| = 0 | 22 | 5.8 (3.4 – 8.3) | 7.654 (0.931 – 62.907) | 0.058 | | | |  | | | |  | | | | |  |  |  |
| *GALNT14*-rs9679162 “TT” |  |  |  | |  | | | | |  | | | |  | | | | | |
| Yes | 12 | 5.3 (3.1 – 7.5) |  |  | | | |  | | | |  | | | | |  |  |  |
| No | 35 | 5.2 (3.5 – 6.9) | 0.847 (0.164 – 4.378) | 0.843 | | | |  | | | |  | | | | |  |  |  |
| *GALNT14*-rs9679162 “GG” |  |  |  | |  | | | | |  | | | |  | | | | | |
| Yes | 8 | 4.8 (1.1 – 8.5) |  |  | | | |  | | | |  | | | | |  |  |  |
| No | 39 | 5.3 (3.8 – 6.8) | 1.864 (0.359 – 9.675) | 0.458 | | | |  | | | |  | | | | |  |  |  |
| *WWOX*-rs13338697 “AA” |  |  |  |  | | | |  | | | |  | | | | |  |  |  |
| Yes | 27 | 5.7 (3.8 – 7.6) |  |  | | | |  | | | |  | | | | |  |  |  |
| No | 20 | 4.7 (2.7 – 6.6) | 0.348 (0.085 – 1.419) | 0.141 | | | |  | | | |  | | | | |  |  |  |
| *WWOX*-rs13338697 “GG” |  |  |  |  | | | |  | | | |  | | | | |  |  |  |
| Yes | 5 | 2.8 (0.1 – 5.6) |  |  | | | |  | | | |  | | | | |  |  |  |
| No | 42 | 5.5 (4.1 – 7.0) | 4.775 (0.493 – 46.271) | 0.177 | | | |  | | | |  | | | | |  |  |  |
| rs6025211 “TT” |  |  |  |  | | | |  | | | |  | | | | |  |  |  |
| Yes | 4 | 3.6 (0.0 – 8.4) |  |  | | | |  | | | |  | | | | |  |  |  |
| No | 43 | 5.4 (4.0 – 6.8) | 3.446 (0.383 – 31.012) | 0.270 | | | |  | | | |  | | | | |  |  |  |
| rs6025211 “CC” |  |  |  |  | | | |  | | | |  | | | | |  |  |  |
| Yes | 18 | 5.4 (3.6 – 7.2) |  |  | | | |  | | | |  | | | | |  |  |  |
| No | 29 | 5.1 (3.2 – 7.1) | 0.540 (0.105 – 2.789) | 0.462 | | | |  | | | |  | | | | |  |  |  |

Median value was used as cutoff for parametric data. The bold indicates the significant P-value. P<0.05 was considered as significant. OS, overall survival; HR, hazard ratio; CI, confidence interval; HBV, hepatitis B virus; HCV, hepatitis C virus; AFP, alpha-fetoprotein; AST, aspartate aminotransferase; ALT, alanine aminotransferase; ALBI, Albumin-bilirubin; ECOG, Eastern Cooperative Oncology Group.

**Table S7.** Univariate and multivariate analysis of clinicopathological factors and SNP genotypes for TTP in 47 ADI-PEG 20 and FOLFOX combination therapy-treated advanced HCC patients.

|  |  | Univariate analysis | | | | | Multivariate analysis | | | | |  |
| --- | --- | --- | --- | --- | --- | --- | --- | --- | --- | --- | --- | --- |
| Parameters | No. patients | Mean TTP (95%CI) | HR (95% CI) | P |  | | | Adjusted HR (95% CI) | | P | | |
| Age (years) |  |  |  |  | |  | | |  | |  |  |
| > 60.3 | 23 | 4.4 (2.7 – 6.0) |  |  | |  | | |  | |  |  |
| ≦ 60.3 | 24 | 5.4 (3.0 – 7.8) | 1.542 (0.669 – 3.556) | 0.309 | |  | | |  | |  |  |
| Gender |  |  |  |  | |  | | |  | |  |  |
| Male | 41 | 4.6 (3.0 – 6.1) |  |  | |  | | |  | |  |  |
| Female | 6 | 7.3 (2.3 – 12.2) | 1.585 (0.467 – 5.378) | 0.460 | |  | | |  | |  |  |
| Tumor number |  |  |  |  | |  | | |  | |  |  |
| > 3 | 13 | 4.6 (2.6 – 6.7) |  |  | |  | | |  | |  |  |
| ≦ 3 | 22 | 4.8 (2.7 – 6.9) | 1.341 (0.543 – 3.311) | 0.524 | |  | | |  | |  |  |
| Largest tumor size (mm) |  |  |  |  | |  | | |  | |  |  |
| > 59.5 | 17 | 4.1 (2.5 – 5.8) |  |  | |  | | |  | |  |  |
| ≦ 59.5 | 17 | 5.2 (2.5 – 7.9) | 0.865 (0.339 – 2.209) | 0.761 | |  | | |  | |  |  |
| AFP (ng/mL) |  |  |  |  | |  | | |  | |  |  |
| > 400.0 | 23 | 3.9 (2.5 – 5.3) |  |  | |  | | |  | |  |  |
| ≦ 400.0 | 16 | 6.2 (3.0 – 9.4) | 1.293 (0.520 – 3.216) | 0.580 | |  | | |  | |  |  |
| Albumin (g/dL) |  |  |  |  | |  | | |  | |  |  |
| > 4.1 | 22 | 4.8 (2.6 – 6.9) |  |  | |  | | |  | |  |  |
| ≦ 4.1 | 25 | 5.0 (3.0 – 7.1) | 1.244 (0.554 – 2.796) | 0.597 | |  | | |  | |  |  |
| Bilirubin (mg/dL) |  |  |  |  | |  | | |  | |  |  |
| > 0.8 | 21 | 4.1 (2.0 – 6.3) |  |  | |  | | |  | |  |  |
| ≦ 0.8 | 26 | 5.5 (3.6 – 7.5) | 1.222 (0.540 – 2.766) | 0.630 | |  | | |  | |  |  |
| Creatinine (mg/dL) |  |  |  |  | |  | | |  | |  |  |
| > 0.9 | 24 | 3.7 (2.1 – 5.3) |  |  | |  | | |  | |  |  |
| ≦ 0.9 | 23 | 6.2 (3.8 – 8.6) | 1.526 (0.661 – 3.521) | 0.322 | |  | | |  | |  |  |
| AST (U/L) |  |  |  |  | |  | | |  | |  |  |
| > 43.5 | 24 | 4.9 (2.9 – 6.9) |  |  | |  | | |  | |  |  |
| ≦ 43.5 | 23 | 4.9 (2.7 – 7.1) | 0.660 (0.292 – 1.492) | 0.318 | |  | | |  | |  |  |
| ALT (U/L) |  |  |  |  | |  | | |  | |  |  |
| > 32.0 | 24 | 4.6 (2.7 – 6.5) |  |  | |  | | |  | |  |  |
| ≦ 32.0 | 23 | 5.3 (3.0 – 7.5) | 0.968 (0.433 – 2.162) | 0.936 | |  | | |  | |  |  |
| Child-Pugh score |  |  |  |  | |  | | |  | |  |  |
| > 5 | 2 | 1.7 (1.2 – 2.1) |  |  | |  | | |  | |  |  |
| ≦ 5 | 45 | 5.1 (3.6 – 6.5) | 0.046 (0.000 – 23801.283) | 0.647 | |  | | |  | |  |  |
| ALBI score |  |  |  |  | |  | | |  | |  |  |
| > -2.8 | 27 | 4.8 (2.9 – 6.7) |  |  | |  | | |  | |  |  |
| ≦ -2.8 | 20 | 5.1 (2.8 – 7.4) | 1.288 (0.559 – 2.967) | 0.552 | |  | | |  | |  |  |
| ALBI grade |  |  |  |  | |  | | |  | |  |  |
| > 1 | 19 | 4.3 (1.8 – 6.9) |  |  | |  | | |  | |  |  |
| ≦ 1 | 28 | 5.3 (3.5 – 7.0) | 0.856 (0.362 – 2.025) | 0.724 | |  | | |  | |  |  |
| ECOG performance status |  |  |  |  | |  | | |  | |  |  |
| = 1 | 25 | 4.7 (3.1 – 6.4) |  |  | |  | | |  | |  |  |
| = 0 | 22 | 5.1 (2.5 – 7.7) | 1.324 (0.572 – 3.065) | 0.512 | |  | | |  | |  |  |
| *GALNT14*-rs9679162 “TT” |  |  |  |  | |  | | |  | |  |  |
| Yes | 12 | 5.0 (2.7 – 7.2) |  |  | |  | | |  | |  |  |
| No | 35 | 4.9 (3.1 – 6.7) | 1.363 (0.570 – 3.255) | 0.486 | |  | | |  | |  |  |
| *GALNT14*-rs9679162 “GG” |  |  |  |  | |  | | |  | |  |  |
| Yes | 8 | 4.3 (0.1 – 8.5) |  |  | |  | | |  | |  |  |
| No | 39 | 5.0 (3.5 – 6.6) | 1.037 (0.350 – 3.074) | 0.948 | |  | | |  | |  |  |
| *WWOX*-rs13338697 “AA” |  |  |  |  | |  | | |  | |  |  |
| Yes | 27 | 5.1 (3.2 – 7.1) |  |  | |  | | |  | |  |  |
| No | 20 | 4.6 (2.4 – 6.9) | 1.049 (0.457 – 2.407) | 0.910 | |  | | |  | |  |  |
| *WWOX*-rs13338697 “GG” |  |  |  |  | |  | | |  | |  |  |
| Yes | 5 | 3.2 (0.0 – 7.3) |  |  | |  | | |  | |  |  |
| No | 42 | 5.1 (3.6 – 6.7) | 1.320 (0.304 – 5.727) | 0.711 | |  | | |  | |  |  |
| rs6025211 “TT” |  |  |  |  | |  | | |  | |  |  |
| Yes | 4 | 3.3 (0.0 – 10.3) |  |  | |  | | |  | |  |  |
| No | 43 | 5.1 (3.6 – 6.6) | 1.957 (0.575 – 6.655) | 0.283 | |  | | |  | |  |  |
| rs6025211 “CC” |  |  |  |  | |  | | |  | |  |  |
| Yes | 18 | 4.8 (3.0 – 6.5) |  |  | |  | | |  | |  |  |
| No | 29 | 5.0 (2.9 – 7.1) | 0.925 (0.399 – 2.144) | 0.855 | |  | | |  | |  |  |

Median value was used as cutoff for parametric data. The bold indicates the significant P-value. P<0.05 was considered as significant. OS, overall survival; HR, hazard ratio; CI, confidence interval; HBV, hepatitis B virus; HCV, hepatitis C virus; AFP, alpha-fetoprotein; AST, aspartate aminotransferase; ALT, alanine aminotransferase; ALBI, Albumin-bilirubin; ECOG, Eastern Cooperative Oncology Group.

**Table S8.** Univariate and multivariate analysis of clinicopathological factors and SNP genotypes for OS in total 160 advanced HCC patients included in this study.

|  |  | Univariate analysis | | | Multivariate analysis | | | |  |
| --- | --- | --- | --- | --- | --- | --- | --- | --- | --- |
| Parameters | No. patients | Mean OS (95%CI) | HR (95% CI) | P |  | Adjusted HR (95% CI) | | P | |
| Age (years) |  |  |  |  |  | |  | | |
| > 60.7 | 80 | 9.1 (7.6 – 10.6) |  |  |  | |  | | |
| ≦ 60.7 | 80 | 9.0 (7.5 – 10.6) | 0.980 (0.687 – 1.399) | 0.912 |  | |  | | |
| Gender |  |  |  |  |  | |  | | |
| Male | 137 | 8.9 (7.8 – 10.0) |  |  |  | |  | | |
| Female | 23 | 10.2 (6.5 – 14.0) | 1.173 (0.701 – 1.963) | 0.544 |  | |  | | |
| Anti-HBV |  |  |  |  |  | |  | | |
| Positive | 80 | 8.4 (6.9 – 9.9) |  |  |  | |  | | |
| Negative | 33 | 9.3 (6.6 – 12.1) | 1.039 (0.675 – 1.602) | 0.861 |  | |  | | |
| Anti-HCV |  |  |  |  |  | |  | | |
| Positive | 29 | 9.6 (6.5 – 12.8) |  |  |  | |  | | |
| Negative | 84 | 8.3 (6.8 – 9.8) | 0.804 (0.506 – 1.278) | 0.356 |  | |  | | |
| Extrahepatic spread |  |  |  |  |  | |  | | |
| Yes | 88 | 8.2 (6.9 – 9.6) |  |  |  | |  | | |
| No | 25 | 10.3 (6.9 – 13.6) | 1.413 (0.862 – 2.316) | 0.170 |  | |  | | |
| Macrovascular invasion |  |  |  |  |  | |  | | |
| Yes | 41 | 7.1 (4.9 – 9.3) |  |  |  | |  | | |
| No | 72 | 9.5 (7.9 – 11.2) | 1.459 (0.966 – 2.205) | 0.073 |  | |  | | |
| Tumor number |  |  |  |  |  | |  | | |
| > 4 | 55 | 6.4 (5.3 – 7.6) |  |  |  | |  | | |
| ≦ 4 | 93 | 10.5 (8.9 – 12.2) | 2.063 (1.412 – 3.014) | **<0.001** |  | |  | | |
| Largest tumor size (mm) |  |  |  |  |  | |  | | |
| > 52.0 | 73 | 8.0 (6.6 – 9.4) |  |  |  | |  | | |
| ≦ 52.0 | 74 | 10.0 (8.2 – 11.8) | 1.221 (0.850 – 1.753) | 0.280 |  | |  | | |
| AFP (ng/mL) |  |  |  |  |  | |  | | |
| > 400.0 | 84 | 7.7 (6.4 – 8.9) |  |  |  | |  | | |
| ≦ 400.0 | 68 | 10.0 (8.2 – 11.9) | 1.392 (0.961 – 2.015) | 0.080 |  | |  | | |
| Albumin (g/dL) |  |  |  |  |  | |  | | |
| > 4.0 | 77 | 9.5 (8.0 – 11.0) |  |  |  | |  | | |
| ≦ 4.0 | 83 | 8.7 (7.1 – 10.2) | 0.896 (0.628 – 1.278) | 0.544 |  | |  | | |
| Bilirubin (mg/dL) |  |  |  |  |  | |  | | |
| > 0.7 | 78 | 8.4 (6.8 – 9.9) |  |  |  | |  | | |
| ≦ 0.7 | 82 | 9.7 (8.2 – 11.3) | 1.165 (0.818 – 1.659) | 0.398 |  | |  | | |
| Creatinine (mg/dL) |  |  |  |  |  | |  | | |
| > 0.8 | 92 | 9.0 (7.7 – 10.4) |  |  |  | |  | | |
| ≦ 0.8 | 68 | 9.1 (7.3 – 10.9) | 1.017 (0.711 – 1.456) | 0.925 |  | |  | | |
| AST (U/L) |  |  |  |  |  | |  | | |
| > 54.0 | 78 | 8.2 (6.7 – 9.7) |  |  |  | |  | | |
| ≦ 54.0 | 82 | 9.9 (8.3 – 11.4) | 1.407 (0.986 – 2.007) | 0.060 |  | |  | | |
| ALT (U/L) |  |  |  |  |  | |  | | |
| > 37.0 | 79 | 9.7 (8.1 – 11.4) |  |  |  | |  | | |
| ≦ 37.0 | 81 | 8.4 (7.0 – 9.9) | 0.886 (0.621 – 1.264) | 0.504 |  | |  | | |
| Child-Pugh score |  |  |  |  |  | |  | | |
| > 5 | 21 | 7.1 (4.2 – 10.1) |  |  |  | |  | | |
| ≦ 5 | 139 | 9.4 (8.2 – 10.6) | 1.273 (0.740 – 2.190) | 0.383 |  | |  | | |
| ALBI score |  |  |  |  |  | |  | | |
| > -2.7 | 72 | 8.3 (6.6 – 10.0) |  |  |  | |  | | |
| ≦ -2.7 | 88 | 9.7 (8.3 – 11.2) | 1.299 (0.910 – 1.854) | 0.150 |  | |  | | |
| ALBI grade |  |  |  |  |  | |  | | |
| > 1 | 63 | 8.8 (6.9 – 10.7) |  |  |  | |  | | |
| ≦ 1 | 97 | 9.3 (7.9 – 10.6) | 1.109 (0.772 – 1.594) | 0.575 |  | |  | | |
| ECOG performance status |  |  |  |  |  | |  | | |
| = 1 | 55 | 8.5 (6.9 – 10.0) |  |  |  | |  | | |
| = 0 | 105 | 9.4 (7.9 – 10.9) | 0.896 (0.608 – 1.321) | 0.580 |  | |  | | |
| *GALNT14*-rs9679162 “TT” |  |  |  |  |  | |  | | |
| Yes | 52 | 9.5 (7.7 – 11.3) |  |  |  | |  | | |
| No | 108 | 8.9 (7.5 – 10.3) | 0.995 (0.655 – 1.391) | 0.809 |  | |  | | |
| *GALNT14*-rs9679162 “GG” |  |  |  |  |  | |  | | |
| Yes | 31 | 9.1 (6.7 – 11.4) |  |  |  | |  | | |
| No | 129 | 9.1 (7.8 – 10.3) | 1.023 (0.658 – 1.590) | 0.920 |  | |  | | |
| *WWOX*-rs13338697 “AA” |  |  |  |  |  | |  | | |
| Yes | 87 | 8.4 (7.0 – 9.8) |  |  |  | |  | | |
| No | 73 | 9.8 (8.1 – 11.6) | 1.096 (0.768 – 1.565) | 0.612 |  | |  | | |
| *WWOX*-rs13338697 “GG” |  |  |  |  |  | |  | | |
| Yes | 14 | 13.4 (7.2 – 19.4) |  |  |  | |  | | |
| No | 146 | 8.7 (7.6 – 9.7) | 0.602 (0.311 – 1.164) | 0.131 |  | |  | | |
| rs6025211 “TT” |  |  |  |  |  | |  | | |
| Yes | 22 | 7.4 (5.0 – 9.8) |  |  |  | |  | | |
| No | 138 | 9.3 (8.1 – 10.5) | 1.560 (0.964 – 2.524) | 0.070 |  | |  | | |
| rs6025211 “CC” |  |  |  |  |  | |  | | |
| Yes | 68 | 10.0 (8.3 – 11.8) |  |  |  | |  | | |
| No | 92 | 8.4 (7.0 – 9.8) | 0.745 (0.518 – 1.071) | 0.112 |  | |  | | |

Median value was used as cutoff for parametric data. The bold indicates the significant P-value. P<0.05 was considered as significant. OS, overall survival; HR, hazard ratio; CI, confidence interval; HBV, hepatitis B virus; HCV, hepatitis C virus; AFP, alpha-fetoprotein; AST, aspartate aminotransferase; ALT, alanine aminotransferase; ALBI, Albumin-bilirubin; ECOG, Eastern Cooperative Oncology Group.

**Table S9.** Univariate and multivariate analysis of clinicopathological factors and SNP genotypes for TTR in total 160 advanced HCC patients included in this study.

|  |  | Univariate analysis | | | | | Multivariate analysis | | | | |  |
| --- | --- | --- | --- | --- | --- | --- | --- | --- | --- | --- | --- | --- |
| Parameters | No. patients | Mean TTR (95%CI) | HR (95% CI) | P |  | | | Adjusted HR (95% CI) | | P | | |
| Age (years) |  |  |  |  | |  | | |  | |  |  |
| > 60.7 | 80 | 4.1 (3.4 – 4.8) |  |  | |  | | |  | |  |  |
| ≦ 60.7 | 80 | 4.1 (3.3 – 4.8) | 1.293 (0.392 – 4.265) | 0.673 | |  | | |  | |  |  |
| Gender |  |  |  |  | |  | | |  | |  |  |
| Male | 137 | 4.0 (3.4 – 4.5) |  |  | |  | | |  | |  |  |
| Female | 23 | 4.5 (3.2 – 5.8) | 0.316 (0.128 – 1.941) | 0.316 | |  | | |  | |  |  |
| Anti-HBV |  |  |  |  | |  | | |  | |  |  |
| Positive | 80 | 3.4 (3.0 – 3.9) |  |  | |  | | |  | |  |  |
| Negative | 33 | 4.0 (2.9 – 5.1) | 37.128 (0.000 – 10261440.2) | 0.572 | |  | | |  | |  |  |
| Anti-HCV |  |  |  |  | |  | | |  | |  |  |
| Positive | 29 | 4.6 (3.2 – 5.9) |  |  | |  | | |  | |  |  |
| Negative | 84 | 3.2 (2.8 – 3.6) | 0.023 (0.000 – 3835.070) | 0.539 | |  | | |  | |  |  |
| Extrahepatic spread |  |  |  |  | |  | | |  | |  |  |
| Yes | 88 | 3.2 (2.8 – 3.6) |  |  | |  | | |  | |  |  |
| No | 25 | 4.8 (3.3 – 6.3) | 0.519 (0.031 – 8.692) | 0.648 | |  | | |  | |  |  |
| Macrovascular invasion |  |  |  |  | |  | | |  | |  |  |
| Yes | 41 | 3.3 (2.4 – 4.2) |  |  | |  | | |  | |  |  |
| No | 72 | 3.8 (3.2 – 4.3) | 2.788 (0.173 – 44.864) | 0.470 | |  | | |  | |  |  |
| Tumor number |  |  |  |  | |  | | |  | |  |  |
| > 4 | 55 | 3.0 (2.6 – 3.5) |  |  | |  | | |  | |  |  |
| ≦ 4 | 93 | 4.4 (3.8 – 5.1) | 0.753 (0.082 – 6.920) | 0.802 | |  | | |  | |  |  |
| Largest tumor size (mm) |  |  |  |  | |  | | |  | |  |  |
| > 52.0 | 73 | 3.6 (3.1 – 4.2) |  |  | |  | | |  | |  |  |
| ≦ 52.0 | 74 | 4.1 (3.4 – 4.8) | 1.238 (0.306 – 5.017) | 0.765 | |  | | |  | |  |  |
| AFP (ng/mL) |  |  |  |  | |  | | |  | |  |  |
| > 400.0 | 84 | 3.7 (3.1 – 4.3) |  |  | |  | | |  | |  |  |
| ≦ 400.0 | 68 | 4.3 (3.4 – 5.2) | 1.246 (0.331 – 4.693) | 0.745 | |  | | |  | |  |  |
| Albumin (g/dL) |  |  |  |  | |  | | |  | |  |  |
| > 4.0 | 77 | 4.3 (3.5 – 5.0) |  |  | |  | | |  | |  |  |
| ≦ 4.0 | 83 | 3.9 (3.2 – 4.6) | 1.071 (0.325 – 3.529) | 0.910 | |  | | |  | |  |  |
| Bilirubin (mg/dL) |  |  |  |  | |  | | |  | |  |  |
| > 0.7 | 78 | 4.1 (3.3 – 4.9) |  |  | |  | | |  | |  |  |
| ≦ 0.7 | 82 | 4.0 (3.4 – 4.7) | 2.270 (0.593 – 8.696) | 0.231 | |  | | |  | |  |  |
| Creatinine (mg/dL) |  |  |  |  | |  | | |  | |  |  |
| > 0.8 | 92 | 4.1 (3.4 – 4.8) |  |  | |  | | |  | |  |  |
| ≦ 0.8 | 68 | 4.1 (3.3 – 4.8) | 0.683 (0.206 – 2.264) | 0.533 | |  | | |  | |  |  |
| AST (U/L) |  |  |  |  | |  | | |  | |  |  |
| > 54.0 | 78 | 3.9 (3.2 – 4.6) |  |  | |  | | |  | |  |  |
| ≦ 54.0 | 82 | 4.2 (3.4 – 5.0) | 0.640 (0.184 – 2.221) | 0.482 | |  | | |  | |  |  |
| ALT (U/L) |  |  |  |  | |  | | |  | |  |  |
| > 37.0 | 79 | 4.1 (3.4 – 4.9) |  |  | |  | | |  | |  |  |
| ≦ 37.0 | 81 | 4.0 (3.3 – 4.7) | 0.511 (0.148 – 1.758) | 0.287 | |  | | |  | |  |  |
| Child-Pugh score |  |  |  |  | |  | | |  | |  |  |
| > 5 | 21 | 3.3 (2.5 – 4.1) |  |  | |  | | |  | |  |  |
| ≦ 5 | 139 | 4.2 (3.6 – 4.8) | 2.951 (0.591 – 14.746) | 0.187 | |  | | |  | |  |  |
| ALBI score |  |  |  |  | |  | | |  | |  |  |
| > -2.7 | 72 | 3.7 (3.0 – 4.5) |  |  | |  | | |  | |  |  |
| ≦ -2.7 | 88 | 4.3 (3.6 – 5.0) | 1.222 (0.371 – 4.027) | 0.742 | |  | | |  | |  |  |
| ALBI grade |  |  |  |  | |  | | |  | |  |  |
| > 1 | 63 | 3.9 (3.0 – 4.7) |  |  | |  | | |  | |  |  |
| ≦ 1 | 97 | 4.2 (3.6 – 4.8) | 1.323 (0.401 – 4.364) | 0.646 | |  | | |  | |  |  |
| ECOG performance status |  |  |  |  | |  | | |  | |  |  |
| = 1 | 55 | 3.8 (3.0 – 4.6) |  |  | |  | | |  | |  |  |
| = 0 | 105 | 4.2 (3.5 – 4.9) | 4.774 (1.229 – 18.534) | **0.024** | |  | | |  | |  |  |
| *GALNT14*-rs9679162 “TT” |  |  |  |  | |  | | |  | |  |  |
| Yes | 52 | 4.0 (3.1 – 4.8) |  |  | |  | | |  | |  |  |
| No | 108 | 4.1 (3.5 – 4.8) | 0.491 (0.106 – 2.275) | 0.363 | |  | | |  | |  |  |
| *GALNT14*-rs9679162 “GG” |  |  |  |  | |  | | |  | |  |  |
| Yes | 31 | 4.1 (3.0 – 5.1) |  |  | |  | | |  | |  |  |
| No | 129 | 4.1 (3.5 – 4.6) | 1.163 (0.244 – 5.538) | 0.849 | |  | | |  | |  |  |
| *WWOX*-rs13338697 “AA” |  |  |  |  | |  | | |  | |  |  |
| Yes | 87 | 4.1 (3.3 – 4.8) |  |  | |  | | |  | |  |  |
| No | 73 | 4.1 (3.4 – 4.7) | 0.461 (0.134 – 1.582) | 0.218 | |  | | |  | |  |  |
| *WWOX*-rs13338697 “GG” |  |  |  |  | |  | | |  | |  |  |
| Yes | 14 | 3.9 (2.3 – 5.5) |  |  | |  | | |  | |  |  |
| No | 146 | 4.0 (3.5 – 4.6) | 3.557 (0.731 – 17.305) | 0.116 | |  | | |  | |  |  |
| rs6025211 “TT” |  |  |  |  | |  | | |  | |  |  |
| Yes | 22 | 2.9 (2.2 – 3.6) |  |  | |  | | |  | |  |  |
| No | 138 | 4.3 (3.7 – 4.8) | 2.133 (0.243 – 18.690) | 0.494 | |  | | |  | |  |  |
| rs6025211 “CC” |  |  |  |  | |  | | |  | |  |  |
| Yes | 68 | 4.4 (3.7 – 5.1) |  |  | |  | | |  | |  |  |
| No | 92 | 3.8 (3.1 – 4.6) | 0.495 (0.130 – 1.887) | 0.303 | |  | | |  | |  |  |

Median value was used as cutoff for parametric data. The bold indicates the significant P-value. P<0.05 was considered as significant. OS, overall survival; HR, hazard ratio; CI, confidence interval; HBV, hepatitis B virus; HCV, hepatitis C virus; AFP, alpha-fetoprotein; AST, aspartate aminotransferase; ALT, alanine aminotransferase; ALBI, Albumin-bilirubin; ECOG, Eastern Cooperative Oncology Group.

**Table S10.** Univariate and multivariate analysis of clinicopathological factors and SNP genotypes for TTP in total 160 advanced HCC patients included in this study.

|  |  | Univariate analysis | | | | | Multivariate analysis | | | | |  |
| --- | --- | --- | --- | --- | --- | --- | --- | --- | --- | --- | --- | --- |
| Parameters | No. patients | Mean TTP (95%CI) | HR (95% CI) | P |  | | | Adjusted HR (95% CI) | | P | | |
| Age (years) |  |  |  |  | |  | | |  | |  |  |
| > 60.7 | 80 | 3.7 (2.9 – 4.5) |  |  | |  | | |  | |  |  |
| ≦ 60.7 | 80 | 3.5 (2.6 – 4.5) | 1.071 (0.734 – 1.564) | 0.721 | |  | | |  | |  |  |
| Gender |  |  |  |  | |  | | |  | |  |  |
| Male | 137 | 3.5 (2.9 – 4.2) |  |  | |  | | |  | |  |  |
| Female | 23 | 4.2 (2.7 – 5.7) | 1.077 (0.649 – 1.788) | 0.774 | |  | | |  | |  |  |
| Anti-HBV |  |  |  |  | |  | | |  | |  |  |
| Positive | 80 | 3.0 (2.3 – 3.7) |  |  | |  | | |  | |  |  |
| Negative | 33 | 3.2 (2.1 – 4.3) | 0.963 (0.613 – 1.514) | 0.870 | |  | | |  | |  |  |
| Anti-HCV |  |  |  |  | |  | | |  | |  |  |
| Positive | 29 | 3.8 (2.5 – 5.2) |  |  | |  | | |  | |  |  |
| Negative | 84 | 2.8 (2.2 – 3.5) | 0.855 (0.535 – 1.366) | 0.512 | |  | | |  | |  |  |
| Extrahepatic spread |  |  |  |  | |  | | |  | |  |  |
| Yes | 88 | 2.7 (2.2 – 3.2) |  |  | |  | | |  | |  |  |
| No | 25 | 4.5 (2.5 – 6.4) | 1.814 (1.059 – 3.105) | **0.030** | | 1.513 (0.850 – 2.692) | | | 0.159 | |  |  |
| Macrovascular invasion |  |  |  |  | |  | | |  | |  |  |
| Yes | 41 | 3.1 (1.7 – 4.4) |  |  | |  | | |  | |  |  |
| No | 72 | 3.1 (2.5 – 3.6) | 1.020 (0.637 – 1.634) | 0.934 | |  | | |  | |  |  |
| Tumor number |  |  |  |  | |  | | |  | |  |  |
| > 4 | 55 | 2.3 (1.8 – 2.8) |  |  | |  | | |  | |  |  |
| ≦ 4 | 93 | 4.2 (3.3 – 5.0) | 1.811 (1.194 – 2.747) | **0.005** | | 1.639 (1.007 – 2.667) | | | **0.047** | |  |  |
| Largest tumor size (mm) |  |  |  |  | |  | | |  | |  |  |
| > 52.0 | 73 | 3.1 (2.5 – 3.8) |  |  | |  | | |  | |  |  |
| ≦ 52.0 | 74 | 3.7 (2.8 – 4.7) | 1.275 (0.866 – 1.879) | 0.219 | |  | | |  | |  |  |
| AFP (ng/mL) |  |  |  |  | |  | | |  | |  |  |
| > 400.0 | 84 | 3.1 (2.4 – 3.7) |  |  | |  | | |  | |  |  |
| ≦ 400.0 | 68 | 4.1 (3.0 – 5.2) | 1.397 (0.948 – 2.057) | 0.091 | |  | | |  | |  |  |
| Albumin (g/dL) |  |  |  |  | |  | | |  | |  |  |
| > 4.0 | 77 | 3.9 (3.0 – 4.8) |  |  | |  | | |  | |  |  |
| ≦ 4.0 | 83 | 3.4 (2.6 – 4.2) | 0.958 (0.660 – 1.391) | 0.823 | |  | | |  | |  |  |
| Bilirubin (mg/dL) |  |  |  |  | |  | | |  | |  |  |
| > 0.7 | 78 | 3.7 (2.7 – 4.7) |  |  | |  | | |  | |  |  |
| ≦ 0.7 | 82 | 3.5 (2.9 – 4.2) | 0.698 (0.473 – 1.028) | 0.069 | |  | | |  | |  |  |
| Creatinine (mg/dL) |  |  |  |  | |  | | |  | |  |  |
| > 0.8 | 92 | 3.7 (2.8 – 4.5) |  |  | |  | | |  | |  |  |
| ≦ 0.8 | 68 | 3.5 (2.7 – 4.4) | 0.878 (0.603 – 1.279) | 0.498 | |  | | |  | |  |  |
| AST (U/L) |  |  |  |  | |  | | |  | |  |  |
| > 54.0 | 78 | 3.4 (2.6 – 4.2) |  |  | |  | | |  | |  |  |
| ≦ 54.0 | 82 | 3.8 (2.9 – 4.7) | 1.076 (0.740 – 1.566) | 0.701 | |  | | |  | |  |  |
| ALT (U/L) |  |  |  |  | |  | | |  | |  |  |
| > 37.0 | 79 | 3.6 (2.7 – 4.4) |  |  | |  | | |  | |  |  |
| ≦ 37.0 | 81 | 3.7 (2.8 – 4.5) | 0.995 (0.684 – 1.448) | 0.980 | |  | | |  | |  |  |
| Child-Pugh score |  |  |  |  | |  | | |  | |  |  |
| > 5 | 21 | 3.3 (1.4 – 5.2) |  |  | |  | | |  | |  |  |
| ≦ 5 | 139 | 3.7 (3.0 – 4.3) | 1.022 (0.580 – 1.801) | 0.940 | |  | | |  | |  |  |
| ALBI score |  |  |  |  | |  | | |  | |  |  |
| > -2.7 | 72 | 3.2 (2.3 – 4.2) |  |  | |  | | |  | |  |  |
| ≦ -2.7 | 88 | 3.9 (3.1 – 4.7) | 0.997 (0.682 – 1.459) | 0.988 | |  | | |  | |  |  |
| ALBI grade |  |  |  |  | |  | | |  | |  |  |
| > 1 | 63 | 3.4 (2.4 – 4.5) |  |  | |  | | |  | |  |  |
| ≦ 1 | 97 | 3.7 (3.0 – 4.5) | 0.914 (0.618 – 1.353) | 0.653 | |  | | |  | |  |  |
| ECOG performance status |  |  |  |  | |  | | |  | |  |  |
| = 1 | 55 | 3.4 (2.5 – 4.3) |  |  | |  | | |  | |  |  |
| = 0 | 105 | 3.7 (2.9 – 4.5) | 0.987 (0.660 – 1.476) | 0.951 | |  | | |  | |  |  |
| *GALNT14*-rs9679162 “TT” |  |  |  |  | |  | | |  | |  |  |
| Yes | 52 | 3.2 (2.3 – 4.1) |  |  | |  | | |  | |  |  |
| No | 108 | 3.8 (3.0 – 4.6) | 1.191 (0.800 – 1.774) | 0.390 | |  | | |  | |  |  |
| *GALNT14*-rs9679162 “GG” |  |  |  |  | |  | | |  | |  |  |
| Yes | 31 | 3.4 (2.2 – 4.6) |  |  | |  | | |  | |  |  |
| No | 129 | 3.7 (3.0 – 4.4) | 1.138 (0.717 – 1.807) | 0.584 | |  | | |  | |  |  |
| *WWOX*-rs13338697 “AA” |  |  |  |  | |  | | |  | |  |  |
| Yes | 87 | 3.4 (2.7 – 4.2) |  |  | |  | | |  | |  |  |
| No | 73 | 3.8 (2.9 – 4.7) | 1.051 (0.723 – 1.527) | 0.795 | |  | | |  | |  |  |
| *WWOX*-rs13338697 “GG” |  |  |  |  | |  | | |  | |  |  |
| Yes | 14 | 4.5 (1.5 – 7.5) |  |  | |  | | |  | |  |  |
| No | 146 | 3.5 (2.9 – 4.1) | 0.775 (0.401 – 1.496) | 0.447 | |  | | |  | |  |  |
| rs6025211 “TT” |  |  |  |  | |  | | |  | |  |  |
| Yes | 22 | 2.2 (1.3 – 3.2) |  |  | |  | | |  | |  |  |
| No | 138 | 3.8 (3.2 – 4.5) | 2.043 (1.207 – 3.425) | **0.008** | | 2.119 (1.171 – 3.837) | | | **0.013** | |  |  |
| rs6025211 “CC” |  |  |  |  | |  | | |  | |  |  |
| Yes | 68 | 3.8 (3.0 – 4.7) |  |  | |  | | |  | |  |  |
| No | 92 | 3.5 (2.6 – 4.3) | 0.956 (0.657 – 1.391) | 0.815 | |  | | |  | |  |  |

Median value was used as cutoff for parametric data. The bold indicates the significant P-value. P<0.05 was considered as significant. OS, overall survival; HR, hazard ratio; CI, confidence interval; HBV, hepatitis B virus; HCV, hepatitis C virus; AFP, alpha-fetoprotein; AST, aspartate aminotransferase; ALT, alanine aminotransferase; ALBI, Albumin-bilirubin; ECOG, Eastern Cooperative Oncology Group.
